# Beta-catenin cleavage enhances transcriptional activation

**DOI:** 10.1038/s41598-017-18421-8

**Published:** 2018-01-12

**Authors:** Tatiana Goretsky, Emily M. Bradford, Qing Ye, Olivia F. Lamping, Tomas Vanagunas, Mary Pat Moyer, Patrick C. Keller, Preetika Sinh, Josep M. Llovet, Tianyan Gao, Qing-Bai She, Linheng Li, Terrence A. Barrett

**Affiliations:** 1 Department of Internal Medicine, Division of Gastroenterology, University of Kentucky, Lexington, KY USA; 2 Markey Cancer Center, University of Kentucky, Lexington, KY USA; 3 Louisiana State University Health Sciences Center, New Orleans, LA USA; 4 INCELL Corporation, San Antonio, TX USA; 5 Northwestern University, Chicago, IL USA; 6 The Mount Sinai Hospital, New York, NY USA; 7 Stowers Institute for Medical Research, Kansas City, MO USA; 8 Dept of Pathology and Laboratory Medicine, University of Kansas Medical Center, Kansas City, KS USA

## Abstract

Nuclear activation of Wnt/β-catenin signaling is required for cell proliferation in inflammation and cancer. Studies from our group indicate that β-catenin activation in colitis and colorectal cancer (CRC) correlates with increased nuclear levels of β-catenin phosphorylated at serine 552 (pβ-Cat^552^). Biochemical analysis of nuclear extracts from cancer biopsies revealed the existence of low molecular weight (LMW) pβ-Cat^552^, increased to the exclusion of full size (FS) forms of β-catenin. LMW β-catenin lacks both termini, leaving residues in the armadillo repeat intact. Further experiments showed that TCF4 predominantly binds LMW pβ-Cat^552^ in the nucleus of inflamed and cancerous cells. Nuclear chromatin bound localization of LMW pβ-Cat^552^ was blocked in cells by inhibition of proteasomal chymotrypsin-like activity but not by other protease inhibitors. K48 polyubiquitinated FS and LMW β-catenin were increased by treatment with bortezomib. Overexpressed *in vitro* double truncated β-catenin increased transcriptional activity, cell proliferation and growth of tumor xenografts compared to FS β-catenin. Serine 552-> alanin substitution abrogated K48 polyubiquitination,  β-catenin nuclear translocation and tumor xenograft growth. These data suggest that a novel proteasome-dependent posttranslational modification of β-catenin enhances transcriptional activation. Discovery of this pathway may be helpful in the development of diagnostic and therapeutic tools in colitis and cancer.

## Introduction

β-catenin is a cytoplasmic protein that participates in intercellular adhesion and Wnt-mediated transcriptional activation (for review see^1^). Wnt/β-catenin - induced gene transcription plays a central role in self-renewal, proliferation, differentiation, polarity, morphogenesis, and development^2–4^. Aberrant Wnt/β-catenin signaling is found in several tumors, including colorectal cancer (CRC)^4,5^. β-catenin signaling is increased in over 90% of CRC due to mutations in either β-catenin exon 3 or adenomatous polyposis coli (APC), believed to enhance β-catenin stability by reducing degradation^6,7^. Ultimately β-catenin translocates into the nucleus and binds transcription factor TCF4 (T cell factor 4) to drive transcription of Wnt regulated genes^6,8–11^.

The primary structure of β-catenin is composed of N and C terminal regions and a central core of 12 armadillo repeats spanning residues 134−678. Cadherins, APC and TCF family transcription factors bind to β-catenin within the core region, whereas GSK3β and α-catenin bind sites within N terminal amino acids^12^. Phosphorylation of N terminal sites targets β-catenin for degradation in the ubiquitin–proteasome pathway in the cytosol^7^. Despite the association of N terminal phosphorylation to degradation, the roles of β-catenin N and C terminal regions to signaling are less clear. Deletion studies indicate that the N terminal domain is not essential for signaling; rather, its absence may enhance stabilization^13^. Studies by Funayama *et al*. indicate that Wnt signaling is increased in Xenopus embryos deficient in the C terminal domain^14^. In other studies by Cox *et al*., data indicate that complete deletion of the C terminus reduces signaling^15,16^. In addition, studies in Drosophila suggest that the C terminal region can be divided into three domains. The region distal to 757 is dispensable for Wnt signaling^14,17,18^. The region between 710 and 757 enhances signaling but is not essential. Lastly, studies by Mo *et al*. indicate that the proximal C terminal regions (also known as the transactivation domain) stabilize β-catenin armadillo repeats making this domain essential for Wnt signaling. Together, these studies indicate that β-catenin regions essential for Wnt signaling include the Armadillo repeat core and proximal C terminal regions (also known as the transactivation domain)^19,20^. Transgenic studies in mice where endogenous β-catenin was replaced by mutant forms with D164A substitution and deleted C-terminus show the ability of this mutant to suppress transcription despite preserved adhesive function^21^. Studies in Drosophila with mutated Armadillo protein (truncated to only 12 armadillo repeats) show increased transcriptional activation after stimulation^22^. In general, studies highlight the importance of Armadillo and C terminal transactivation domains in β-catenin signaling while suggesting that other regions are dispensible.

In humans and rodents, increased PI3K (phosphatidylinositide 3-kinase) activation seen with diminished PTEN (phosphatase and tensin homolog) levels enhances β-catenin activation. Studies from He *et al*. indicate that Akt phosphorylates β-catenin at serine 552 located in the open loop within the tenth Armadillo repeat. In PTEN mutant mice (Mx1Cre/PTEN^fl/fl^), increased numbers of epithelial cells with nuclear phospho-β-catenin^Ser552^ (pβ-Cat^552^) were detected in small bowel polyps. Staining for pβ-Cat^552^ co-localized with stem cell markers (Msi-1, phospho-PTEN, β-Gal) and increased TCF4 transcriptional activity seen in Mx1Cre/PTEN^fl/fl^xTOP-GAL mice^23^. Using an antibody specific for pβ-Cat^552^, we detected enhanced nuclear staining in epithelial cells in human colitis and colorectal cancer^24^. Given that anti-pβ-Cat^552^ staining identifies an epitope with the core region, we further explored its role in activation of β-catenin signaling.

Studies here interrogate alterations in β-catenin that occur during Wnt/β-catenin signaling in cancer and mucosal inflammation. The data reveal that post-translational modifications of β-catenin in the ubiquitin-proteasome pathway yield a truncated β-catenin molecule containing a serine 552-phosphorylated core region without N and C termini. This proteolytic processing of β-catenin is required for binding with TCF4 and subsequent transcriptional activation.

## Results

### Low-molecular weight β-catenin predominates in the nuclei of cancer cells

**Figure 1:**
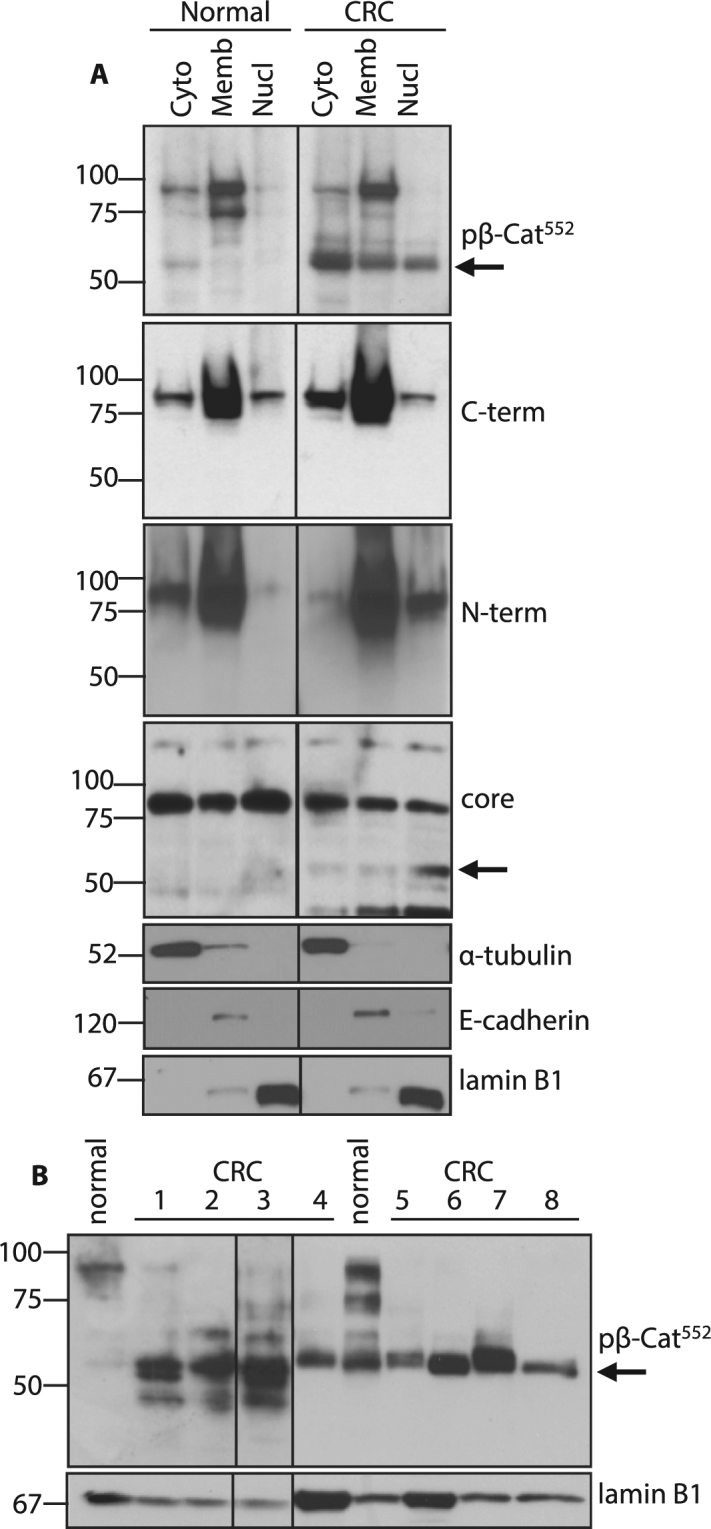
Nuclear low-molecular weight (LMW) β-catenin predominates in colon cancer IEC. Protein isolates from biopsy-derived normal and colorectal cancer (CRC) IEC were probed with antibodies specific for distinct β-catenin epitopes. (**A**) Normal and CRC IEC fraction lysates (cytosolic (Cyto), membrane (Memb) and nuclear (Nucl) were probed sequentially for β-catenin antibodies directed to different epitopes as shown. Purity controls were achieved by probing for α-tubulin (cytosol), E-cadherin (membrane) and lamin B1 (nucleus). (**B**) Nuclear lysates from normal and CRC biopsies were probed for phospho-β-catenin^Ser552^ (pβ-Cat^552^). WB for lamin B1 serves as loading control. Arrows indicate positions of LMW β-catenin. Full size membrane scans for WBs can be seen in Suppl. Fig. SS1.

Mutations in components of the destruction complex (e.g. APC, β-catenin, Axin) are associated with increased nuclear accumulation of β-catenin and enhanced Wnt/β-catenin signaling in tumor cells^5^. To examine the molecular events occurring during enhanced β-catenin signaling in the colon, intestinal epithelial cell (IEC) nuclear isolates from patients with CRC and colitis were assayed by WB (western blot) using antibodies specific for β-catenin epitopes at N and C termini as well as within the region of armadillo repeats (core and pβ-Cat^552^). Data in Fig. 1A indicate that full-size (FS) (~86-90 kD) β-catenin was detected by antibodies specific for N-terminal, C-terminal, core region (armadillo repeats) and pβ-Cat^552^ epitopes in cytosolic and membrane fractions. Examination of nuclear fractions indicates that levels of FS β-catenin detected by C-terminal and core region-specific antibodies were similar in normal and CRC tissue. N-terminal antibody detected enhanced levels of β-catenin in this sample, while other WB failed to detect increased N-terminal β-catenin (Suppl. Fig. S5A) suggesting this finding was inconsistent.

Interestingly, a smaller, 52–56 kD fragment of β-catenin detected by core region and pβ-Cat^552^antibodies was increased in CRC tissue compared to normal (arrows). Given that both core and pβ-Cat^552^antibodies identify epitopes within the armadillo repeats, we postulated that β-catenin may be cleaved into a low molecular weight (LMW) form in transformed cells.

To confirm the presence of LWM β-catenin in other CRC tissues, we surveyed epithelial tissue from sporadic CRC as well as metastatic lesions in the liver from primary CRC tumors. Data show that anti-pβ-Cat^52^ antibody detected FS β-catenin in normal colon compared to predominantly LMW β- catenin present in relatively high levels in CRC. In most cases, the LMW form of nuclear β-catenin detected by anti-pβ-Cat^552^ antibody was found to the exclusion of higher molecular weight forms (Fig. 1B). This pattern was evident in tumors from all examined regions of the colon (we had tested more than fifty individual biopsies). Examination of liver tissue that harbored metastatic lesions from CRC revealed that LMW nuclear pβ-Cat^552^ predominated in hepatic metastasis compared to adjacent normal liver where FS β-catenin was seen (Suppl. Fig. S1A). Furthermore, relatively high levels of LWM β-catenin were detected by the anti-pβ-Cat^552^ antibody in lung, pancreas and primary liver tumor tissues compared to matched normal tissues (Suppl. Fig. S1B). In cancer-bearing cirrhotic livers, LMW β-catenin levels were greater in tumor (hepatoma) compared to adjacent cirrhotic tissue. We also found that nuclear levels of LMW β-catenin were increased in colitis compared to normal IEC and that levels were clearly enriched in colitis-associated cancers (CAC) (Suppl. Fig. S1C and D). Of note, LMW β-catenin was present in anti-CD45 depleted IEC but not CD45-sorted cell fractions (Suppl. Fig. S1E). Together these data indicate that increased levels of LMW pβ-Cat^552^ are associated with enhanced epithelial Wnt/β-catenin signaling as seen in cancer and colitis.

To confirm the specificity of antibodies detecting LMW β-catenin, siRNA knockdown of β-catenin in normal human colonic epithelial cells (NCM460) was utilized. As shown in Suppl. Fig. S2B and C LMW bands of β-catenin detected with core and pβ-Cat^552^ specific antibodies were significantly reduced after siRNA (small (or short) interfering RNA) treatment. Together, these results indicate that antibodies to core and pβ-Cat^552^ epitopes identify a LMW form of β-catenin present in normal cells and increased in states of enhanced Wnt signaling.

### Nuclear appearance of LMW β-catenin is proteasome-dependent

**Figure 2:**
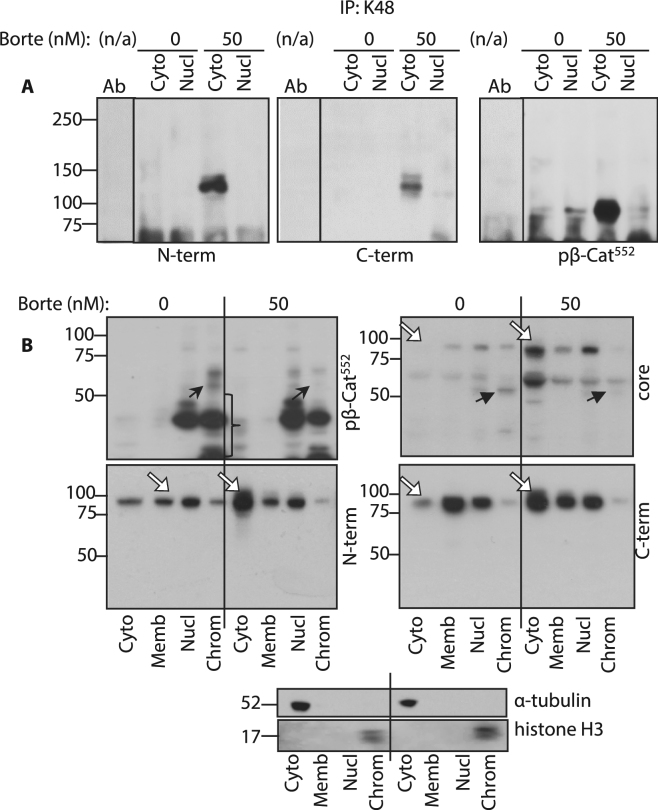
β-catenin truncation is proteasome dependent. (**A**) Cytosolic and nuclear lysates from HT29 cells, treated with bortezomib (Borte) were immunoprecipitated for lysine 48 polyubiquitin chain (K48) and probed for N, C and pβ-Cat^552^ epitopes of β-catenin. (**B**) HT29 cells treated with bortezomib were fractionated to cytosolic (Cyto), membranous (Memb), soluble (Nucl) and chromatin-bound (Chrom) nuclear fractions. WBs were run sequentially for β-catenin antibodies specific for epitopes as indicated. Solid arrows depict decreases in LMW β-catenin with bortezomib. Open arrows depict increases in full length β-catenin after bortezomib treatment. The braces indicate pβ-Cat^552^ likely processed in the nuclear proteasome. α-tubulin is a loading and purity control for cytosolic fraction, histone H3 - for chromatin bound nuclear fraction. Full size membrane scans for WBs can be seen in Suppl. Fig. SS2.

To examine potential mechanisms for generating LMW β-catenin, we tested the hypothesis that β-catenin cleavage was dependent on cytosolic proteasome activity. As an attachment of lysine 48 (K48) polyubiquitin chain (poly-Ub) has been shown to target proteins to the proteasome for degradation^25,26^, we examined its role in β-catenin processing. Cytosolic and nuclear fractions of colon cancer cell line HT29 were used for immunoprecipitation (IP) with an antibody specific for the K48 poly-Ub and then probed for β-catenin epitopes within N and C termini as well as pβ-Cat^552^. Data in Fig. 2A show that the proteasome inhibitor bortezomib^27^ increased levels of K48 ubiquitinated β-catenin in the cytosol. As expected, N and C terminus antibodies detected molecular forms of poly-Ub-β-catenin at MW 120 kD. These findings were consistent with the predicted molecular weight (MW) of β-catenin (88 kD) and attached side chain composed of four ubiquitins (8.5 kD×4 = 34 kD). By comparison, the antibody specific for pβ-Cat^552^ recognized poly-Ub-β-catenin of approximately 86–90 kD MW. These data were consistent with the combined MW of LMW β-catenin (52–56 kD) and four ubiquitins (34 kD). The detection of K48 polyubiquitinated FS and LMW pβ-Cat^552^ in bortezomib-treated samples suggests that both FS and LMW pβ-Cat^552^ are degraded in the proteasome following K48 polyubiquitination. In one of the repeated experiments with HT29 cells (as done in Fig. 2A) we detected an increase in poly-Ub-pβ-Cat^552^ at 120 kD (seen in over-exposed WB, blue arrow, at Suppl. Fig. S3A). Interesting, a notable amount of ubiquitinated LMW pβ-Cat^552^ is detected in nuclear soluble fractions (red arrow at Suppl. Fig. S3A). These findings support our hypothesis that serine 552  phosphorylation at FS and LMW β-catenin is a signal for K48 ubiquitination (confirmed by experiments in RKO cells transfected with mutated serine 552 -> alanin (Ser552->Ala) β-catenin constructs, which will be discussed below) and suggest that further degradation of β-catenin may occur in the nucleus. In addition, we found that proteasome inhibition greatly reduced chromatin-bound β-catenin levels, especially LMW forms detected by anti-core region and pβ-Cat^552^ antibodies (Fig. 2B). In all cases, proteasome inhibition increased cytosolic levels of FS β-catenin (Fig. 2B). In Suppl. Fig. S3B are shown results of K48 IP of NCM460 cells after treatment with siRNA to β-catenin. The nearly complete attenuation of bands identified by C terminal and anti-pβ-Cat^552^ antibodies confirms that these derive from β-catenin. To confirm that β-catenin antibodies detected proteins covalently bound to the ubiquitin chain, denatured proteins were precipitated with anti-K48 antibody and probed with N-terminal and pβ-Cat^552^ antibodies (Suppl. Fig. S3C). WBs revealed bands with the same MW detected in Fig. 2A.

**Figure 3:**
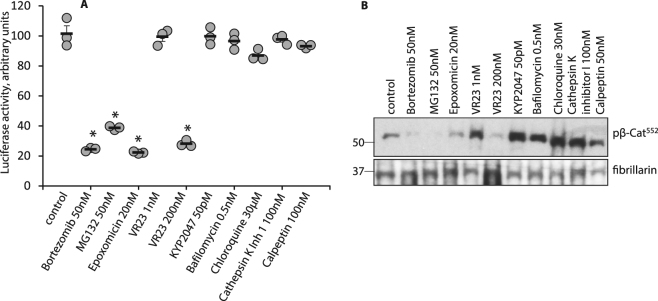
Inhibitors of chymotrypsin-like activity of the proteasome diminish β-catenin transcriptional activity. (**A**) HT29 cells transfected with TCF/LEF reporter were treated with inhibitors as indicated. Luciferase activity is shown in cells incubated with protease inhibitors. Asterisks indicate statistically significant p values compared to control. p < 0.001. (**B**) HT29 cells were treated as in **A** and nuclear chromatin-bound fractions probed for pβ-Cat^552^. Fibrillarin served as a loading control. Full size membrane scans for WBs can be seen  in Suppl. Fig. SS3.

To address whether the proteolytic cleavage of β-catenin reported here occurs due to a regulated process, we first identified proteases with the potential to target β-catenin (see: http://web.expasy.org/peptide_cutter)^28^. Next, a number of inhibitors specific for relevant proteases were used to treat HT29 colon cancer cells. Data show that inhibition of chymotrypsin-like activity with epoxomicin^29^, bortezomib, VR23^30^ (200 nM), and MG312^31^ reduced TCF/LEF (T cell factor 4/lymphoid enhancer-binding factor) luciferase activity (Fig. 3A) as well as levels of LMW pβ-Cat^552^ in chromatin-bound nuclear fractions (Fig. 3B). By comparison, inhibitors of proteasome trypsin-like activity (VR23, 1 nM), proline endopeptidase (KYP2047), lysosomal endopeptidases (bafilomycin, chloroquine), cathepsin K or proteinase K (calpeptin) failed to affect TCF/LEF luciferase activity (Fig. 3A) or LMW pβ-Cat^552^ levels. These findings support the conclusion that β-catenin is specifically cleaved by chymotrypsin-like activity within the proteasome.

To address possible concerns that LMW β-catenin bands seen on WBs are the result of proteasome activity after cell lysis, human biopsies from normal and colitic patients were divided in half and fractionated into cytosolic, membranous and nuclear protein lysates. One half was fractionated in the presence of epoxomicin. As shown on Suppl. Fig. S4, addition of the proteasome inhibitor failed to alter pβ-Cat^552^ levels detected by WB.

### TCF4 binds LMW-β-catenin

**Figure 4:**
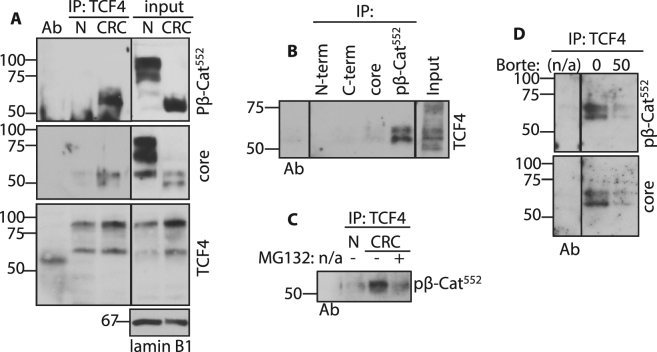
TCF4 binds proteasome-sensitive LMW-β-catenin. (**A**) IEC nuclear fractions from normal (N) and CRC biopsies were immunoprecipitated by anti-TCF4 and probed for pβ-Cat^552^, core region β-catenin and TCF4. (**B**) Nuclear fractions from CRC biopsies were used for IP with different β-catenin antibodies. Precipitated proteins were probed with anti-TCF4 antibody. Control WBs with N and C terminal, core region specific and pβ-Cat^552^ antibodies can be seen in Suppl. Fig. S6D. (**C**) Normal, CRC and CRC treated with MG132 IEC nuclear fraction were immunoprecipitated by anti-TCF4 and probed for pβ-Cat^552^. Input and loading control WBs for these samples can be seen on Suppl. Fig. S6E. (**D**) Chromatin-bound nuclear fractions of HT29 cell, untreated and treated with bortezomib (input and loading controls in Fig. 2B), were immunoprecipitated by anti-TCF4 and probed for anti-pβ-Cat^552^ and core region β-catenin antibodies. Full size membrane scans for WBs can be seen at Suppl. Fig. SS4.

As we detected multiple molecular weight forms of nuclear β-catenin, we next examined β-catenin binding to TCF4. Proteins immunoprecipitated by anti-TCF4 were probed with different β-catenin antibodies. Antibodies specific for pβ-Cat^52^ and core region detected β-catenin/TCF4 binding predominantly in CRC, but not antibodies for N and C terminal β-catenin epitopes (Fig. 4A and Suppl. Fig. S6A). Reverse co-IPs support these data, as the pβ-Cat^552^ antibody precipitated TCF4 (Fig. 4B). The two bands of TCF4 seen in Fig. 4B may be explained by recent observations from Weise *et al*. that alternative splicing of TCF4 transcripts generates protein variants (M1/S2)^32^. In CRC biopsy samples treated with proteasome inhibitor MG132 *ex vivo*, as well as in HT29 cells treated with bortezomib, we found that proteasome inhibition greatly reduced nuclear levels of pβ-Cat^552^ bound to TCF4 (Fig. 4C and D). The effect of proteasome inhibition on above mentioned TCF4 transcriptional activity was supported by results in TCF/LEF luciferase reporter assays in HT29 (Fig. 3A) and NCM460 cell lines (Suppl. Fig. S5).

### Tumor necrosis factor (TNF), Wnt and carcinogenic transformation upregulate chromatin-bound LMW-β-catenin

**Figure 5:**
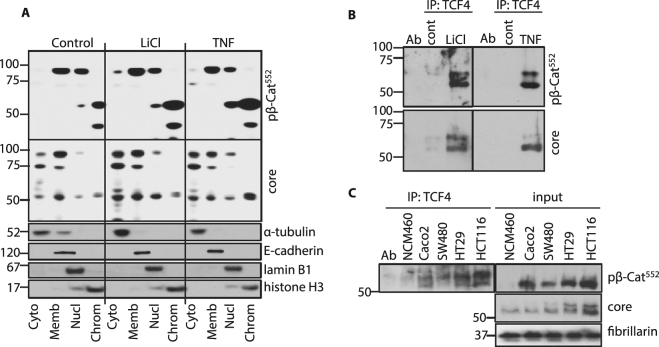
TNF, LiCl and carcinogenic transformation increase abundance of chromatin-bound LMW-β-catenin and TCF4 binding. (**A**) NCM460 cells were treated with LiCl and TNF and fractionated as in Fig. 2B. WBs were probed sequentially with anti-pβ-Cat^552^ and anti-core region β-catenin antibodies. WBs for α-tubulin, e-cadherin, laminB1 and histone H3 represent loading controls in sub-cellular fractions. (**B**) Chromatin-bound fractions from (**A**) were immunoprecipitated by anti-TCF4 and probed for pβ-Cat^552^ and core region specific antibodies. (**C**) Chromatin-bound fractions from NCM460 and indicated colon cancer cell lines were immunoprecipitated by anti-TCF4 and probed for pβ-Cat^552^. Input control WB was also probed for core region β-catenin and fibrillarin. Full size membrane scans for WBs can be seen in Suppl. Fig. SS5.

As data in Suppl. Fig. S1C indicate that IEC nuclear β-catenin levels increase in IBD, we examined nuclear accumulation of β-catenin following TNF treatment. Cytosolic, membranous, nuclear soluble and chromatin-bound fractions were isolated from NCM460 cells and probed for LMW β-catenin. Data revealed that the majority of LMW β-catenin associates with the chromatin-bound protein fraction as identified by core region and pβ-Cat^552^ antibodies (Fig. 5A). Both LiCl and TNF upregulate chromatin-bound levels of LMW β-catenin and TCF4/β-catenin binding in chromatin-bound protein extracts (Fig. 5B). Taken together, these data indicate that canonical Wnt signaling and inflammatory cytokines induce β-catenin binding to TCF4 in the chromatin-bound fraction of nuclear extracts. WBs of nuclear extracts immunoprecipitated with anti-TCF4 also revealed that LEF-1 binding increased with TNF (Suppl. Fig. S6B). Significantly increased binding of pβ-Cat^552^ to TCF4 was also detected in colon cancer cell lines with different mutations in β-catenin signaling pathway - Caco2, SW480, HT29 and HCT116 (Fig. 5D).

To confirm that bands detected after TCF4 IP belong to β-catenin we used nuclear extracts from NCM460 cells treated with siRNA to β-catenin and stimulated with TNF (Suppl. Fig. S6C). The significant attenuation of bands identified by anti-core and anti-pβ-Cat^552^ antibodies confirms that these derive from β-catenin.

To examine the association of LMW β-catenin with active Wnt signaling in normal colonic stem cells, we utilized a well-characterized model of *in vitro* colonic stem cell expansion published by Hans Clevers and colleagues^33^. In these cultures, growth of colonic crypt epithelial cells under high Wnt (Methods) conditions promotes expression of stem cell genes whereas low Wnt (Methods) conditions inhibit stem cell expansion/gene transcription. In data presented in Suppl. Fig. S7A and B, we show that colonoids grown under high Wnt conditions are noticeably larger and express increased mRNA (message RNA) for genes associated with colonic epithelial stem cells (Lgr5, Axin2, CD44, PCNA) compared to colonoids grown under low Wnt conditions. WB results of pβ-Cat^552^ show greater levels of pβ-Cat^552^ localized to chromatin-bound fractions in cells grown under high Wnt compared to low Wnt conditions (Suppl. Fig. S7C). Probing WBs with an antibody specific for C terminal β-catenin revealed that cells grown in high Wnt had lower levels of FS β-catenin compared to cells grown in low Wnt. The absence of C terminal β-catenin in chromatin-bound fractions of either low Wnt or high Wnt colonoids was consistent with the notion that the C terminus was cleaved from the β-catenin detected in chromatin-bound fractions (Suppl. Fig. S7C, upper panel) with anti-pβ-Cat^552^.

### Overexpressed double truncated β-catenin increases β-catenin signaling in NCM460 cells

**Figure 6:**
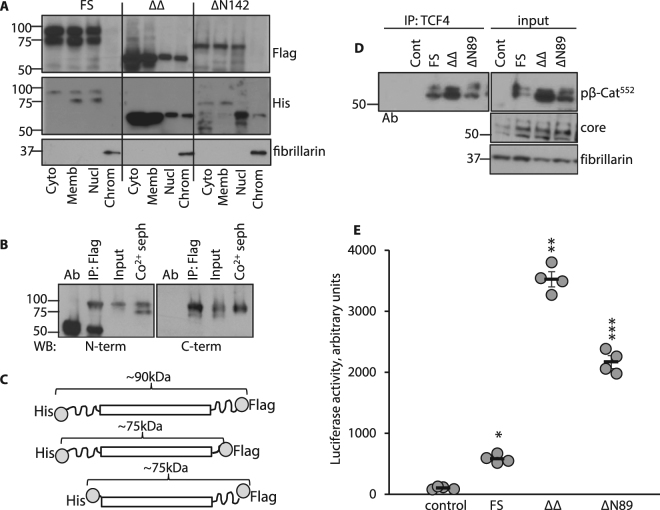
Overexpressed double truncated β-catenin increases transcriptional activity. (**A**) NCM460 cells were infected with FS, ∆∆ and ∆N142 β-catenin constructs tagged with His (N terminus) and Flag (C terminus). WBs were probed sequentially with anti-Flag and anti-His antibodies. Fibrillarin served as loading and purity controls for chromatin bound fractions. (**B**) Total cell lysates of NCM460 cells overexpressing FS β-catenin were precipitated with anti-Flag antibody or (Co^2+^) sepharose (His tag specific). Proteins were resolved on SDS PAGE and WBs probed sequentially with N and C termini specific anti-β-catenin antibodies. (**C**) Schematic representation of β-catenin constructs used in (**A** and **B**). (**D**) Chromatin bound fractions from NCM460 cells are shown: vector control, FS, ∆∆ and ∆N89 β-catenin overexpressing cell lines immunoprecipitated with anti-TCF4 and probed with pβ-Cat^552^ and core region antibodies. Fibrillarin serves as a loading control for input. (**E**) FS, ∆∆ and ∆N89 β-catenin overexpressing NCM460 cell lines were co-transfected with TCF/LEF reporter plasmid and β-catenin-induced luciferase activities measured. *p = 0.002, **p = 0.001, ***p = 0.003. Flag WB of total cell lysates used in (**D** and **E**) (to evaluate levels of overexpressed β-catenin) can be seen on Suppl. Fig. S8C. Full size membrane scans for WBs can be seen  in Suppl. Fig. SS6.

Given findings that nuclear LMW β-catenin levels were increased in colon, pancreas, lung and liver tumors, we suspected that protein cleavage was associated with β-catenin transcriptional activity. To test this notion, NCM460 cells were transfected with constructs encoding FS β-catenin, and β-catenin truncated at N and C termini. The “double truncated” β-catenin, referred to as ∆∆ β-catenin was generated based on the predicted chymotrypsin cutting sites outside of armadillo repeats (see: http://web.expasy.org/peptide_cutter)^28^. From the total of 28 possible sites flanking N and C termini of the armadillo repeats, we choose high specificity sites  tyrosin142 and  phenylalanin 683. To test if treatment with chymotrypsin  would generate peptides with molecular weight close to 52–56 kDa we used recombinant β-catenin. As seen on Suppl. Fig. S8A overnight treatment with chymotrypsin yielded fragments close to this molecular weight. Thus, ∆∆ β-catenin contained amino acids 143 to 683 of β-catenin. The ∆N142 protein includes the armadillo sequences along with an intact C-terminus (amino acids 143 to 781). All constructs were tagged with His at the N-terminus and Flag at the C-terminus (Fig. 6C). Results in Fig. 6A indicate that Flag and His-tagged proteins were detected in cytosolic, membrane and nuclear soluble fractions of cells transfected with FS, ∆∆ and ∆N142 β-catenin constructs. However, examination of chromatin-bound fractions revealed significant differences in detection patterns of Flag and His-tagged proteins among transfected cells. First, Flag and His-tagged proteins were not detected in chromatin-bound fractions of cells transfected with FS β-catenin. Secondly, LMW His-labeled proteins, but not Flag-tagged proteins, were detected in chromatin-bound fractions of cells transfected with the ∆N142 construct suggesting that cleavage of C terminal sequences occurred prior to translocation. Lastly, detection of both Flag and His-tagged proteins in chromatin-bound fractions of ∆∆ β-catenin transfected cells suggested this protein localized without further cleavage. These data were confirmed by cobalt (Co^2+^) sepharose pull down (His tag specific) experiment presented on Suppl. Fig. S8B, where only ∆∆ β-catenin Flag tagged protein was detected in chromatin-bound fraction but not FS or ∆N142.

Interestingly, there were 75kDa bands seen in the FS panel identified by both Flag and His tags. We propose these bands represent β-catenin fragments where the N terminus has been cleaved (identified by Flag tag) or where the C-terminal sequence has been cleaved (identified by His tag). Both of the residual peptides weighed approximately 75kD. To confirm these interpretations, we performed pull down experiments in which total cell lysates were immunoprecipitated with anti-Flag antibody or precipitated with His tag specific Co^2+^ sepharose beads. Sequential WBs with N and C terminus specific antibodies (Fig. 6B) revealed full size (~90kD) peptides and those where cleavage of C or N termini resulted in 75kD fragments. The remaining peptides contained armadillo repeats with N or C termini respectively. A single  ~90kD band was detected in anti-Flag IP probed with N terminal Ab whereas two bands were seen in His specific sepharose beads precipitates probed with N terminal antibody (~90 and ~75kD). (A diagram of these peptides is shown in Fig. 6C). On the right panel of Fig. 6B, Flag IP of lysates were probed with C terminal  antibody. Again we see full size β-catenin (88 kD) along with a ~75kD fragment containing armadillo and C terminal peptides. In the lane on the right panel of Fig. 6B showing His specific Co^2+^ sepharose precipitate, the C terminal WB shows a single ~90kD full size band without a ~75 kD band containing C terminal sequences because this peptide was absent (see diagram in Fig. 6C). These latter data were consistent with the interpretation that the N terminal peptide containing His tag was lost after cleavage leaving an intermediate fragment containing armadillo and C terminal (Flag-tagged) peptides. The data provide further support for the notion that N and C terminal regions of β-catenin are sequentially cleaved prior to nuclear translocation.

To examine the effect of β-catenin truncation on TCF4 binding and transcriptional activation, NCM460 cells were transfected with FS, ∆∆ and ∆N89 β-catenin  (a molecule with first 89  amino acids truncated). Cells were transfected with ∆N89 β-catenin to allow comparisons to mutated, “constitutively active” β-catenin^13,34,35^. TCF4 IP studies showed that levels of pβ-Cat^552^ bound to TCF4 were highest in ∆∆β-catenin cells compared to ∆N89 or FS β-catenin (Fig. 6D). Similarly, TCF4/LEF luciferase activity was highest in cells transfected with ∆∆ compared to ∆N89 or FS β-catenin (Fig. 6E). Total cell lysate WBs (Suppl. Fig. S8C) show equal expression of tagged β-catenin in the cell lines used in Fig. 6D and E.

To investigate the ability of ∆∆ β-catenin to increase expression of β-catenin target genes, mRNA expression from NCM460 transfected with FS and ∆∆ β-catenin was compared to vehicle control. As presented in Suppl. Fig. S8D elevated levels of β-catenin target gene mRNA (Axin2, Lgr5, CD44, ASCL2, Ki97 and cyclin D1) were detected in cells overexpressing ∆∆ β-catenin. c-myc mRNA expression wasn’t changed. Interestingly, increases in c-myc and cyclin D1 protein levels (WB) were greater (Suppl. Fig. 8E) than mRNA changes. This might reflect an importance of post-translational regulation of c-myc and cyclin D1 expression by reduced degradation of c-myc and nuclear accumulation of cyclin D1. c-myc protein is degraded after K48 ubiquitination in normal cell, while in cancer it is stabilized and accumulated in cytosol^36^. Alterations in cyclin D1 turnover in cancer can lead to nuclear accumulation of cyclin D1 independent of changes in cyclin D1 mRNA expression. In normal, cyclin D1 protein is translocated in cytosol where it is utilized by proteasome^37^.

Together, these data indicate that LMW β-catenin efficiently localizes to chromatin-bound fractions where it binds TCF4 and drives TCF/LEF transcriptional activity.

### Double truncation and serine 552 phosphorylation of β-catenin increases tumor invasiveness

To further investigate whether β-catenin truncation conveys enhanced cell invasiveness, NCM460 cells transfected with control, FS β-catenin, ∆N89 β-catenin and ∆∆β-catenin vectors were examined in methylcellulose colony formation and proliferation assays. Findings in Suppl. Fig. S9A and B show that cells transfected with ∆∆ β-catenin invaded and proliferated at higher levels compared to those transfected with ∆N89 or FS constructs. These findings suggest that double truncated β-catenin promotes cellular responses associated with tumor invasiveness.

**Figure 7:**
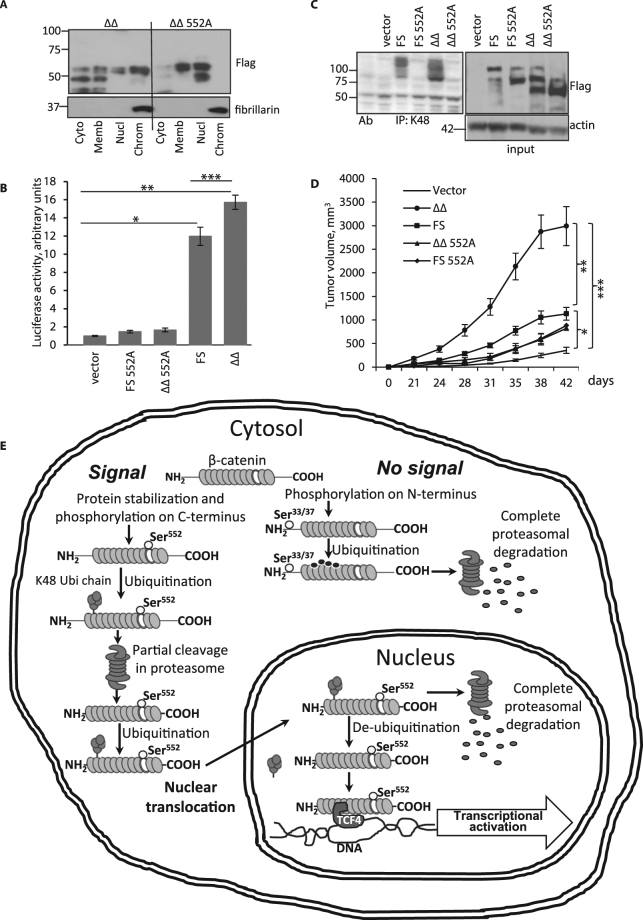
β-catenin serine 552 phosphorylation enhances translocation to chromatin-bound fraction and increases xenograft tumor growth. (**A**) RKO cells were transfected with FS, ∆∆, FS 552A and ∆∆ 552A β-catenin and fractionated. WBs were probed with anti-Flag antibody. Fibrillarin served as loading and purity controls for chromatin-bound fractions. (**B**) RKO cell lines overexpressing wild type  and mutated β-catenin were co-transfected with TCF/LEF reporter plasmid and luciferase assay performed. *p = 0.002, **p = 0.001, ***p = 0.005. (**C**) RKO cells were treated with 20nM epoxomicin and cytosolic lysates precipitated with K48 specific antibody. WBs were developed with Flag tag antibody. Actin served as a loading control. (**D**) Xenograft mice were injected with RKO cells transfected with control and overexpressing β-catenin constructs. The graph represents volumes of developed tumors. *p = 0.016; **p = 0.04; ***p = 0.003. n = 7. Full size membrane scans for WBs can be seen in Suppl. Fig. SS7. (**E**) Proposed mechanism of β-catenin transcriptional activation.

Phosphorylation of β-catenin at serine 552 has been reported to affect nuclear translocation^23,38^. To examine the impact of β-catenin  serine 552 phosphorylation on localization to chromatin bound fractions, RKO tumor cells were transfected with distinct ∆∆ β-catenin constructs containing wild type (FS and ΔΔ) or Ser552->Ala substituted (FS 552A and ∆∆ 552A) sequences. RKO colon cancer cells were used to avoid mutations in the β-catenin signaling pathway. All constructs were Flag tagged at the C terminus. As seen in Fig. 7A, Flag tagged ∆∆ β-catenin was prevalent in cytosol, membrane, nuclear and chromatin-bound fractions whereas Flag tagged ∆∆552A was detected in membrane and nuclear fractions without appreciable levels seen in cytosol or chromatin-bound fractions suggesting serine 552 phosphorylation was needed for efficient localization to nuclear chromatin. Analyses of TCF/LEF transcriptional activity showed that FS and ∆∆ β-catenin transfected cells exhibited 12 and 16-fold higher luciferase activities, respectively, compared to vector-only controls or alanine-substituted FS 552A or ∆∆ 552A expressing cells (Fig. 7B). Thus, chromatin localization of pβ-Cat^552^ correlated with transcriptional activity.

To examine the impact of serine 552 phosphorylation on β-catenin ubiquitination in the cytosol, RKO cells (transfected as in Fig. 7B) were treated with epoxomicin to block cytosolic proteasome activity. Probing of anti-K48 poly-Ub IPs revealed substantial levels of Flag tagged wild type FS and ∆∆  proteins. In contrast, cells transfected with serine 552 substituted sequences (FS 552A or ∆∆ 552A) failed to yield ubiquitin-bound proteins in epoxomicin treated cells (Fig. 7C). Taken together with other data presented here (Fig. 2A, Suppl. Fig. S3A) these findings suggest that serine 552 phosphorylation promotes ubiquitination and targets β-catenin to the cytosolic proteasome for specific cleavage prior to nuclear translocation (Fig. 7E).

To examine the ability of ∆∆ β-catenin and  serine 552 phosphorylation to promote tumor growth, we utilized a xenograft mouse model. RKO cells transfected with vector, FS, ∆∆, FS 552A and ∆∆ 552A were injected into nude mice. Data presented in Fig. 7D show that RKO cells transfected with ∆∆ β-catenin grew larger than RKO cells transfected with wild type FS or Ser552->Ala - mutated FS or ∆∆ constructs. Colony formation and proliferation assays (Suppl. Fig. S10A–C) confirmed significant advantages of overexpressed ∆∆ β-catenin compared to wild type FS or mutated constructs. Together, these findings support the notion that β-catenin serine 552 phosphorylation and cleavage at both N and C termini promote tumor aggressiveness. In the cartoon model in Fig. 7E we propose a sequence of β-catenin transcriptional activation based on our findings in this study. In this model, activation induces phosphorylation at FS β-catenin serine 552 prior to ubiquitination (with K48 poly-Ubi) and partial cleavage in the cytosolic proteasome. Truncated β-catenin is ubiquitinated again with K48 poly-Ubi and translocated into nucleus. In the nucleus, de-ubiquitinated LMW pβ-Cat^552^ binds TCF4 in chromatin-bound fractions to activate transcription. We further suspect that the remaining ubiquitinated LMW pβ-Cat^552^ goes to the nuclear proteasome for complete degradation.

## Discussion

Data presented here are consistent with a novel view of β-catenin processing during Wnt-activated signaling. In states, where Wnt/β-catenin signaling is increased (colorectal cancer, pancreatic cancer, lung cancer, liver cancer and colitis), we found increased levels of a serine 552 phophorylated LMW form of β-catenin in the nucleus suggesting that β-catenin processing had occurred. Biochemical studies revealed that FS β-catenin was ubiquitinated in the cytosol by K48 poly-chain (Ub-β-catenin) (Figs 2A and 7C) prior to cleavage. The failure to detect LMW β-catenin by antibodies directed against residues in N or C termini suggests that β-catenin undergoes partial site-directed cleavage outside the core armadillo repeat region. The detection of FS pβ-Cat^552^ in the membrane and cytosol but not the nucleus (Figs 1A and 6A) suggests that the cleavage event occurs prior to nuclear translocation. Specific enzyme inhibition studies indicated that chymotrypsin-like activity within the cytosolic proteasome was responsible for β-catenin cleavage (Fig. 3). Detection of LMW poly-Ub pβ-Cat^552^ in bortezomib-treated cells suggests that serine 552 phosphorylation may be a signal for a second step of K48 poly-ubiquitination. This view was supported by studies showing Ser552->Ala mutated β-catenin was not ubiquitinated (Fig. 7C). The failure to detect LMW pβ-cat^552^ in the chromatin bound nuclear fraction of cells treated with bortezomib (Fig. 2B) suggests that K48 ubiquitinated LMW pβ-Cat^552^ was degraded in the proteasome as was FS β-catenin. The ubiquitinated FS β-catenin form is likely retained in the cytosol and unable to translocate to the nucleus. (This process may be analogous to other signaling pathways such as NFkB^39^). In NFκB signaling, the inhibitor IκBα is degraded in the proteasome which frees NF-κB to translocate into the nucleus and regulate gene transcription^40^). In contrast, ubiquitinated ∆∆ β-catenin might translocate in the nucleus, where we propose de-ubiquitinating enzymes generate LMW pβ-Cat^552^ that localizes to the chromatin-bound fraction (Figs 2B, 6A and 7A). Once activated, LMW pβ-Cat^552^ binds TCF4 and drives transcription in chromatin-bound fractions (Figs 5,6 and Suppl. Fig. S6–S8). It is also possible that K48 poly-ubiquitinated pβ-Cat^552^ is degraded in the nucleus by the nuclear proteasome (Fig. 2B, marked with a brace, Suppl. Fig. 3A). We diagramed this proposed model in the cartoon in Fig. 7E.

The detection of relatively high levels of nuclear LMW pβ-Cat^552^ in colon cancer cells suggests that this pathway does not preclude the well-characterized degradation pathway altered by mutations in APC and β-catenin. In these cells, a high level of “stabilized” full length β-catenin is available for phosphorylation and cleavage in the pathway presented here (Figs 5D and 7E). In fact, overexpression of the LMW pβ-Cat^552^ led to higher levels of TCF/LEF luciferase activity (Fig. 6E) and tumor growth (Fig. 7D) suggesting this form may induce higher levels of β-catenin induced transcription in transformed cells. Together these data suggest a novel step in β-catenin signaling involving post-translational modifications (phosphorylation and cleavage). Although levels of LMW pβ-Cat^552^ were pronounced in transformed cells, detection of LMW pβ-Cat^552^ in colitis, high Wnt colonoids and TNF treated normal cell line indicated that β-catenin cleavage likely occurs in non-transformed cells as well (Suppl. Fig. S1C,D; Fig. 5, Suppl. Fig. S7C).

The cytosolic proteasome has been linked to β-catenin degradation following phosphorylation of serine 45 by CK1 (facilitated by APC) and then phosphorylation of serine 33/37 by GSK3β^41^. N terminal phosphorylation targets β-catenin for ubiquitination by E3 ligase which directs Ub-β-catenin to the proteasome^7,42^. Data regarding which type of ubiquitin modification targets β-catenin for degradation under this condition  are limited. Most likely, it is mono-ubiquitination, as shown by Aberle *et al.*^7^. Data presented here indicate that K48 poly-ubiquitination targets FS β-catenin and LMW pβ-Cat^552^ for degradation (partial cleavage) in the cytosolic proteasome (Fig. 2 and 7C). FS pβ-Cat^552^ was detected in the cytosol (Figs 1, 5, Suppl. Fig. S7C) as was LMW pβ-Cat^552^. We did not detect FS pβ-Cat^552^ in chromatin-bound fractions (Figs 5A, 6A, Suppl. Fig. S7C and S8B) suggesting that it is cleaved prior to binding TCF4. The detection of chromatin and TCF4-bound LMW pβ-Cat^552^ but not FS β-catenin is consistent with the notion that LMW pβ-Cat^552^ translocates to nuclear chromatin after cleavage in the cytosol (Fig. 7A).

The findings reported here support the conclusion that β-catenin undergoes an important cleavage step essential for signaling in the nucleus. This event appears to be most prevalent in transformed tissue where β-catenin activation is increased related to mutations in the canonical Wnt/β-catenin pathway (APC, β-catenin, Axin, etc.). The present findings unveil an important nuance of this pathway, that β-catenin undergoes a significant modification on its way to nuclear translocation and TCF4 binding. Careful studies were conducted to support the conclusion that generation of LMW β-catenin resulted from regulated chymotrypsin-like activity in the proteasome (Fig. 3). Given the importance of this pathway in cancer development and metastasis, these findings may be helpful in the development of novel diagnostic and therapeutic tools in colitis and cancer. These data also enhance understanding of mechanisms operating in benign disorders associated with increased β-catenin signaling such as human colitis.

## Materials and Methods

The antibodies used are listed in Suppl. Table S1.

Reagents used: calyculin A (Calbiochem, San Diego, CA); bortezomib and VR23 (Selleck Chemicals, Houston, TX); TNF (Peprotech, Rocky Hill, NJ); MG132, epoxomicin, bafilomycin, chloroquine, cathepsin K inhibitor I, calpeptin, KYP2047 and polybrene (Sigma, St. Louis, MO); zeocin and puromycin (Invitrogen, Carlsbad, CA); siPORT NeoFX transfection reagent (Thermo, Rockford, IL); jetPRIME transfection reagent (Polyplus, New York, NY); luciferase reagent (Promega, Madisson, WI); recombinamt β-catenin (Abcam, Cambridge, MA, cat # ab63175).

### Human biopsy samples

For all human studies, informed consent was obtained from every patient and samples were coded. Collection of all material was approved by the Northwestern University or University of Kentucky Institutional Review Boards, in accordance with their guidelines and regulations. Human colonic biopsy specimens were obtained from patients undergoing diagnostic or surveillance colonoscopy for known or suspected ulcerative colitis (UC) or cancer. Specimens were collected from Northwestern Memorial Hospital (Chicago) and the University of Kentucky and Good Samaritan Hospitals (Lexington, KY). For comparison and *ex vivo* stimulation, biopsy specimens were obtained from healthy patients undergoing routine colon cancer surveillance. Colorectal cancer (CRC) specimens were obtained from patients undergoing surgery. Collection of all patient materials for this study was approved by Institutional Review Board protocol (IRB #13–0559-F3R). Pancreas and lung biopsy samples were obtained from Northwestern Memorial Hospital (Chicago). Liver biopsy samples were homogenized in the laboratory of Professor Josep Llovet and semi-purified protein frozen at −80 °C delivered overnight in dry ice.

### Human biopsy epithelial cell isolation

Human colon epithelia samples were delivered from the operating room in ice cold PBS. Samples were washed once with ice cold PBS and incubated at 4°C with rotation in PBS with 10mM DDT and 50nM calyculin A for 30min, 4°C. The samples were centrifuged at 300 rpm for 5 min. Cells were frozen in liquid nitrogen and stored at −80°C until use.

### Experiment *ex-vivo*

Human colon biopsies from CRC patients were treated as above. The tissue was divided into two parts and treated with MG132 (in HBSS) as indicated (Fig. 4C). Samples were incubated at 4°C with slow rotation for 2hrs, centrifuged at 300 rpm for 5min and supernatant removed. Samples were frozen and stored at −80°C until use.

### Animals

All animal experiments were approved by the University of Kentucky Institutional Animal Care and Use Committee and were conducted in accordance with their regulations and guidelines. Male athymic nude mice (5–6 weeks old) were purchased from Taconic (Hudson, NY, USA). To establishe CRC xenografts, mice were subcutaneously injected with tumor cells (1 × 10^6^/mouse) in a 1:1 mixture of media and Matrigel (Corning Inc., Corning, NY)  (n = 7 per group). Tumor dimensions were measured using a caliper and tumor volumes were calculated as mm^3^ = π/6×(larger diameter)×(smaller diameter)^2,43^.

### Cell culture

NCM460 cells (normal derived colon mucosa cells) were received by a cell licensing agreement with INCELL Corporation (San Antonio, TX), and were routinely propagated under standard conditions in M3:10A medium supplemented by 10% of fetal bovine serum (FBS) with addition of the conditioned medium (33%) from previously cultured NCM460 cells^44^. Cells were treated overnight with 1ng/ml of TNF or with 20mM of LiCl, harvested the next morning and fractionated. For experiments in Suppl. Fig. S6, bortezomib was added at 50nM for 8 hrs.

SW480 cells (ATCC, CCL-228) were cultured in Leibovitz’s L-15 medium with 10% FBS under standard conditions. HT29 (ATCC, HTB-38), RKO (ATCC, CRL-2577), Caco2 (ATCC, HTB-37) and HCT116 (ATCC, CCL-247) cells were cultured in Dulbecco’s Modified Eagle’s Medium (DMEM) with 10% FBS under standard conditions. HT29 cells in *log* phase were treated with a series of specific inhibitors of proteasomal chymotrypsin-like activity (epoxomicin, 20nM; bortezomib, 50nM; VR23, 200nM; and MG312, 50nM, trypsin-like activity of the proteasome (VR23, 1nM), proline endopeptidase (KYP2047, 50nM), lysosomal endopeptidases (bafilomycin, 0.5nM; chloroquine, 30μM), inhibitors of cathepsin K, 100nM, and proteinase K (calpeptin, 100nM). The cells were incubated under standard conditions for 8 hrs with proteasome inhibitors and overnight with other inhibitors. Cells were harvested and fractionated.

### siRNA

siRNA to human β-catenin was obtained from Ambion (Thermo, Rockford, IL; siRNA ID: s438). siRNA was introduced in NCM460 cells with siPORT NeoFX transfection reagent. Forty eight hours after transfection cells were harvested and used for experiments. siRNA nonsense mix (Santa Cruz, Dallas, TX) was used as a control.

### Lentiviral constructs and transductions

The pLV lentiviral plasmids encoding FS, ∆89 β-catenin, ∆N142 and ∆∆ β-catenin with His tag on N terminus and Flag tag on C-terminus were made based on human β-catenin pcDNA3.1 neo (gift from Dr. Eric Fearon: Addgene plasmid # 16828)^31^ with primers listed in Suppl. Table S2. The constructs obtained were cloned into pLV-EF1a-MCS-IRES-GFP-Puro (pLV) (Biosettia Inc. San Diego, CA). ∆N89 pcDNA3.1 neo β-catenin with C terminal Flag tag was a gift from Eric Fearon (Addgene plasmid # 19288)^34^. The insert was also re-cloned into a pLV vector. Primers are listed in Suppl. Table S2. VSV-G pseudotyped lentivirus stocks were made in the DNA/RNA Delivery Core, SDRC (Chicago, IL). NCM460 cells were infected in the presence of 1μg/ml polybrene (Sigma St. Louis, MO) and stable cell lines generated. Cells were maintained under selection pressure with 5μg/ml of puromycin. Expression levels of FS and ∆∆  β-catenin in infected NCM460 cells were assessed by WB for Flag tag.

The reporter construct containing TCF/LEF luciferase (TCF/luc) was generated in the DNA/RNA Delivery Core, SDRC at Northwestern University (Chicago, IL) by inserting six copies of the TCF/LEF response element in the lentiviral pGF1 vector (System Bioscience, Mountain View, CA). The cells were infected with TCF/luc viral particles as described above. Luciferase activity was detected with luciferase reagent.

### pcDNA3.1zeo β-catenin expressing constructs and RKO cells transfections

Wild type FS β-catenin insertion was re-cloned in pcDNA3.1 with zeocin resistance from pcDNA3.1 expressing C terminal FLAG tagged FS β-catenin (gift from Dr. Eric Fearon: Addgene plasmid # 16828)^34^ and pcDNA3.1 expressing C terminal FLAG tagged FS β-catenin with serine 552 substituted to alanine (FS 552A) (gift from Dr. Dexing Fang^38^). Double truncated (∆∆ and ∆∆ 552A) and ∆N142 β-catenin constructs were created with primers listed in Suppl. Table S2. RKO cells were transfected with vector or with β-catenin constructs by using jetPRIME transfection reagent according to the manufacturer’s instructions (Polyplus, New York, NY). Stable cell lines were established under selection pressure of 400 μg/ml of zeocin.

### NCM460 cell proliferation assays

Proliferation assays were performed by using the CyQUANT® Cell Proliferation Assay Kit (Invitrogen, Carlsbad, CA) according to the manufacturer’s instructions.

### RKO cell proliferation assays

Cells were placed in 96 well plates at 10^4^ per well (6 wells for each cell line). Cells were stained with trypan blue and viable cells were counted on a hemocytometer 5 times every 24hrs after initial 48hrs of incubation.

### Colonoids

Colonic crypts were isolated from C57BL\6 mice via collagenase digestion and embedded in Matrigel as previously described^45^. Colonoids were established in WENR media (100ng/ml Wnt3a and 1μg/ml R-spondin)^33^. After 48hrs, media were changed to reduced Wnt3a and R-spondin (25ng/ml Wnt3a and 250ng/ml R-spondin) for 5 days.

### Subcellular protein fractionation

The subcellular protein fractionation (human epithelial cells) protocol was modified from described procedures^46^. All buffers used contained Protease Arrest™ protease inhibitor cocktail (G-Biosciences, St. Louis, MO), and phosphatase inhibitor cocktail I and II (Sigma, St. Louis, MO) at 1:100. Human epithelial cells were homogenized in glass homogenizer in buffer I (50mM Tris-HCl pH 7.4, 100mM NaCl, 0.01% digitonin), lysates were passed through a 250μm tissue strainers (Thermo, Rockford, IL), centrifuged at 4°C for 10 min at maximum speed in table top centrifuge. The supernatants were collected and used as the cytosolic fraction. Pellets were re-suspended in buffer II (50mM Tris-HCl pH 7.4, 2% Triton X100, 100mM NaCl) and incubated on ice for 30min, then centrifuged as above. The supernatants were used as the membrane/organelle fraction. Pellets were dissolved in buffer III (50mM Tris-HCl pH 7.4, 0.25% DDM (n-Dodecyl-D-maltoside), 100mM NaCl), and with 2U of Benzonase (Sigma, St. Louis, MO) per 100µl of lysate and incubated for 30 min at room temperature (RT). Following centrifugation the supernatants were used as nuclear fractions.

Cultured cells and colonoids were fractionated according to Pierce manufacturer’s protocol (Subcellular Protein Fractionation Kit, Thermo, Rockford, IL). In particular, chromatin-bound protein extraction was performed after nuclear soluble proteins isolation. Nuclear Extraction Buffer (NEB-company formulation) with 5μl of 100mM CaCl_2_ and 3μl of micrococcal nuclease (300 units) per 100μl, supplemented with protease and phosphatase inhibitors, was added to the pellet. After vortex for 15sec at highest setting mixture was incubated at room temperature (RT) for 15min and centrifuged at 16,000×*g* (highest setting of microcentrifuge) for 5min. Supernatant was collected and used as chromatin bound fraction of nuclear proteins.

Protein concentration was measured by BCA assay (Thermo, Rockford, IL).

The purity of the fractions was confirmed by WB with anti-α-tubulin, anti-e-cadherin, anti-laminB1, anti-histoneH3, and anti-fibrillarin antibodies (see Suppl. Table S1).

### Immunoprecipitation and western blotting

500µg of nuclear soluble protein or 200µg chromatin-bound protein fractions, 2µg of TCF4 primary antibody coupled to agarose beads (Pierce Co-IP Kit, Thermo, Rockford, IL) were added in each IP reaction and the mixture was incubated overnight at 4^o^C. The beads were washed according to Pierce protocol and the proteins were eluted from agarose, acetone precipitated, and resolved on SDS-PAGE followed by WB.

 In experiments presented in Fig. 2A and in Fig. 4B, 500 µg of cytosolic and nuclear soluble protein or 300 µg of chromatin-bound protein and 2μg of K48 or β-catenin antibodies (β-catenin antibodies were coupled to agarose beads as anti-TCF4 (see above)) were used for each IP reaction. The mixture was incubated overnight at 4°C. 20µl of Protein A/G Plus agarose (Santa Cruz, Dallas, TX) were added to the mixture and incubation continued for another 30min at 4°C with gentle rotation. Agarose beads were washed four times with ice cold RIPA buffer (20% in PBS) and re-suspended in LDS NuPAGE sample buffer (Invitrogen, Carlsbad, CA) with 10% 2-mercaptoethanol. The samples were boiled and resolved with SDS-PAGE, followed by WB.

For WB, proteins were transferred on Immobilon FL (Millipore, Billerica, MS) by semi-dry transfer (Bio-Rad, Hercules, CA) and membranes blocked in Pierce Protein-Free T20 blocking buffer (Thermo, Rockford, IL) for 1hr, and incubated overnight at 4°C in 1:1000 primary antibody solution. Membranes were extensively washed, incubated in 0.02μg/ml secondary antibody for 1hr., washed again and developed using West Pico, Dura or Femto reagent (Thermo, Rockford, IL).

***Cobalt sepharose pulldown*** of total lysates and chromatin-bound fractions from FS, ∆142 and ∆∆ β-catenin overexpressing NCM460 cell lines was performed according to manufacturer’s protocol (Thermo, Rockford, IL). For each reaction 500μg of lysate and 20μl of sepharose were used. Precipitates were run on SDS-PAGE and WB probed with anti-Flag antibody.

### Denatured protein IP

1mg of cytosolic or nuclear protein lysate was used to precipitate with 1:4(v:v) of 100% trichloroacetic acid. After 10min incubation at 4°C samples were centrifuged and pellets were washed with ice-cold acetone. The pellets were dried, reconstituted in RIPA buffer and protein concentration measured by BCA assay. IP was performed as above.

All WB and IP experiments were repeated at least three times.

### Real time PCR

Total RNA was isolated from colonoids or cultured cells using the RNeasy Mini Kit (Qiagen, Valencia, CA) and reverse transcribed using High Capacity cDNA Reverse Transcription Kit (Applied Biosystems, Foster City, CA). Real time PCR used the ABI Step OnePlus real-time PCR system and Power SYBR green PCR master mix (Applied Biosystems). Primers were designed by Primer Express software 3.0 (Applied Biosystems) based on nucleotide sequences from the National Center for Biotechnology Information data bank (Suppl. Table S3). For each sample, glyceraldehyde-3-phosphate dehydrogenase was used as the internal reference. All assays were performed in triplicate and fold changes were calculated using the ΔΔCT method.

### Chymotrypsin cleavage of recombinant β-catenin

DDT was added (final concentration of 5mM) to 5µg of recombinant β-catenin dissolved in 25% glycerol, 50mM Tris-HCl, 150mM NaCl, 0.25mM DDT pH7.5. Protein was incubated 20min at 50–60 °C for 20 min. Reduced protein mixture was cooled to RT and iodoacetamide was added to a final concentration of 15mM. Mixture was Incubated in dark for 15min at RT. Volume was adjusted to 50µl with 100mM Tris-HCl, 10mM CaCl_2_ (pH 8.0) and 2ng/µl chymotrypsin, sequencing grade (Promega, Madison, WI) added to sample. After overnight incubation at 25°C protein was precipitated with acetone (1:10 v/v, 30min at −20°C) and resolved at SDS-PAGE along with intact 5µg of β-catenin. Gel was stained with silver according manufacturer protocol (Fast Silver Kit, G-Bioscience, St. Louis, MO).

### Statistical analysis

All experiments were performed at least three times. Results are expressed as mean ± S.E.M. where applicable. A two-tailed Student’s t-test was used to compare the intergroup. Differences between groups were considered statistically significant at p<0.05.

### Data availability

All data generated or analyzed during this study are included in this published article (and its Suppl. Information files).

## Electronic supplementary material

Supplemental materials
