# Supplementary material for: Beta-catenin cleavage enhances transcriptional activation
Source: Sci Rep. 2018 Jan 12;8:671. doi: 10.1038/s41598-017-18421-8 (PMC5766502; doi:10.1038/s41598-017-18421-8)

Beta-catenin cleavage enhances transcriptional activation.

Tatiana Goretsky[1], Emily M. Bradford[1], Qing Ye[2], Olivia F. Lamping[1],Tomas Vanagunas[3], Mary Pat Moyer[4], Patrick C. Keller[1], Preetika Sinh[5], Josep M. Llovet[6], Tianyan Gao[2], Qing-Bai She[2], Linheng Li[7,8],Terrence A. Barrett[1].

[1]Department of Internal Medicine, Division of Gastroenterology, University of Kentucky, Lexington, KY

[2]Markey Cancer Center, University of Kentucky, Lexington, KY

[3]Louisiana State University Health Sciences Center, New Orleans, LA

[4]INCELL Corporation, San Antonio, TX

[5]Northwestern University, Chicago, IL

[6]The Mount Sinai Hospital, New York, NY

[7]Stowers Institute for Medical Research, Department of Pathology & Laboratory Medicine, The University of Kansas School of Medicine, Kansas City, KS

[8]Dept of Pathology and Laboratory Medicine, University of Kansas Medical Center, Kansas City, KS

Supplemental materials.

Supplemental Table S1. Antibodies used in the study..

**Specificity**	**Vendor, catalog number**
α-tubulin	Sigma, F1804
β-catenin (core region specific)	Abcam, ab64944
β-catenin (core region specific)	Abcam, ab19450 (discontinued)
β-catenin (C-terminus)	BD, 610154
β-catenin (N-terminus)	Santa Cruz, sc-69764
β-actin	Sigma, A3854
E-cadherin	Cell Signaling, 3195
fibrillarin	Santa Cruz, sc-374022
Flag	Abcam, ab1257
His	Santa Cruz, sc-803
HistoneH3	Millipore, 06-755
Lamin B1	Zimed, 33-2000
Polyubiquitin K48	Millipore, 05-1307
pβ-catenin Ser552	Cell signaling, 9566
pβ-catenin Ser552	Custom*
TCF4	Santa Cruz, sc-166699

Supplemental Table S2. β-catenin cloning primers.

Primer name	Primer sequence
FS BamH1 His forward	5' – ATTGGATCCATGCATCATCATCATCACCACGCTACTCAAGCTGATTTGATGGAGTTGGACATGGCC
ΔΔ BamH1 His forward	5' – ATTGGATCCATGCATCATCATCATCACCACCATATCAAGATGATGCAGAACTTGCCACACGTGCAATCCCT
FS Mlu1 Flag reverse	5' – ATTACGCGTTTACTTGTCGTCATCATCCTTGTAATCCAGGTCAGTATCAAACCAGGCCAGCTGATTGCTGTC
ΔΔ Mlu1 Flag reverse	5' – ATTACGCGTTTACTTGTCGTCATCATCCTTGTAATCCTGAAGAGAGAGCTGGTCAGCTCAACTGAAAGCCGTTT
ΔΔ BamH1 forward	5' – ATTGGATCCATGCATATCAAGATGATGCAGAACTTGCCACACGTGCAATCCCT
ΔΔ Not1 Flag reverse	5' – ATTGCGGCCGCTTACTTGTCGTCATCATCCTTGTAATCCTGAAGAGAGAGCTGGTCA

Supplemental Table S3. Real-time PCR primers.

	Forward	Reverse
mGAPDH	GAACGGATTTGGCCGTATTG	TGAGTGGAGTCATACTGGAACATGT
mLGR5	CAAACTTCCCAGAGCTCAAGATTAT	TGTTGCCGTCGTCTTTATTCC
mAXIN2	CGCCAACGACAGCGAGTTAT	CCATCTACGCTACTGTCCGTCAT
mPCNA	TAAAGAAGAGGAGGCGGTAA	TAAGTGTCCCATGTCAGCAA
hGAPDH	GAAGGTGAAGGTCGGAGTC	GAAGATGGTGATGGGATTTC
hAXIN2	GTGTGAGGTCCACGGAAACT	ACAGGATCGCTCCTCTTGAA
hLGR5	TATGACCTGCCTCCCAGTTC	ATGCCACAGAGGAAAGATGG
hCD44	AGCAGCGGCTCCACCATCGAGA	TCGGATCCATGAGTCACAGTG
hC-MYC	GCTGCTTAGACGCTGGATTT	TAACGTTGAGGGGCATCG
hCYCLIND1	ACAAACAGATCATCCGCAAACAC	TGTTGGGGCTCCTCAGGTTC

*He, X. C. et al. PTEN-deficient intestinal stem cells initiate intestinal polyposis. Nat Genet 39, 189-198, (2007).

Supplemental Figures.

Supplemental Figure S1.

Low-molecular weight β-catenin predominates in the nucleus of cancer and colitic cells.

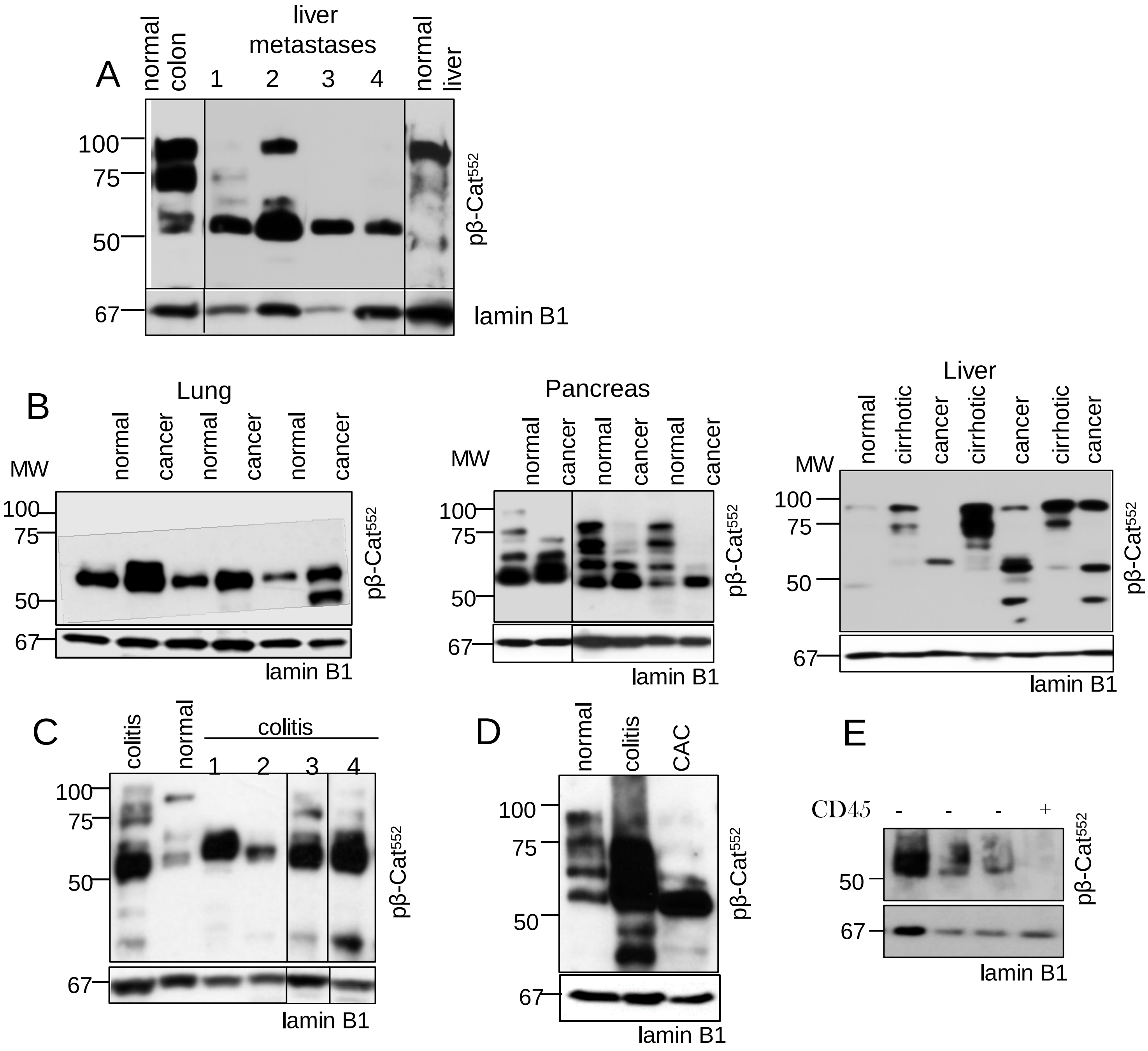

A. Nuclear lysates of biopsies from normal colon and liver and from colorectal cancer metastases in liver probed for pβ-Cat552. ***B.*** Nuclear lysates of lung and pancreas normal and cancer biopsies, liver normal, cirrhotic and cancer, probed for pβ-Cat552. ***C.*** Nuclear lysates of normal and colitis biopsies probed for pβ-Cat522. ***D.*** Normal, colitis and CAC biopsies from the same patient probed for pβ-Cat552. Lamin B1 served as a loading control. ***E.*** Nuclear lysates from colitic biopsy. Lane 1 represents isolates purified by the regular protocol. Second and third lanes are CD45 negative epithelial cell fractions. Lane 4 shows the pool of CD45 positive cells. Lamin B1 served as a loading control.

Supplemental Figure S2.

LMW β-catenin is knocked down with specific siRNA.

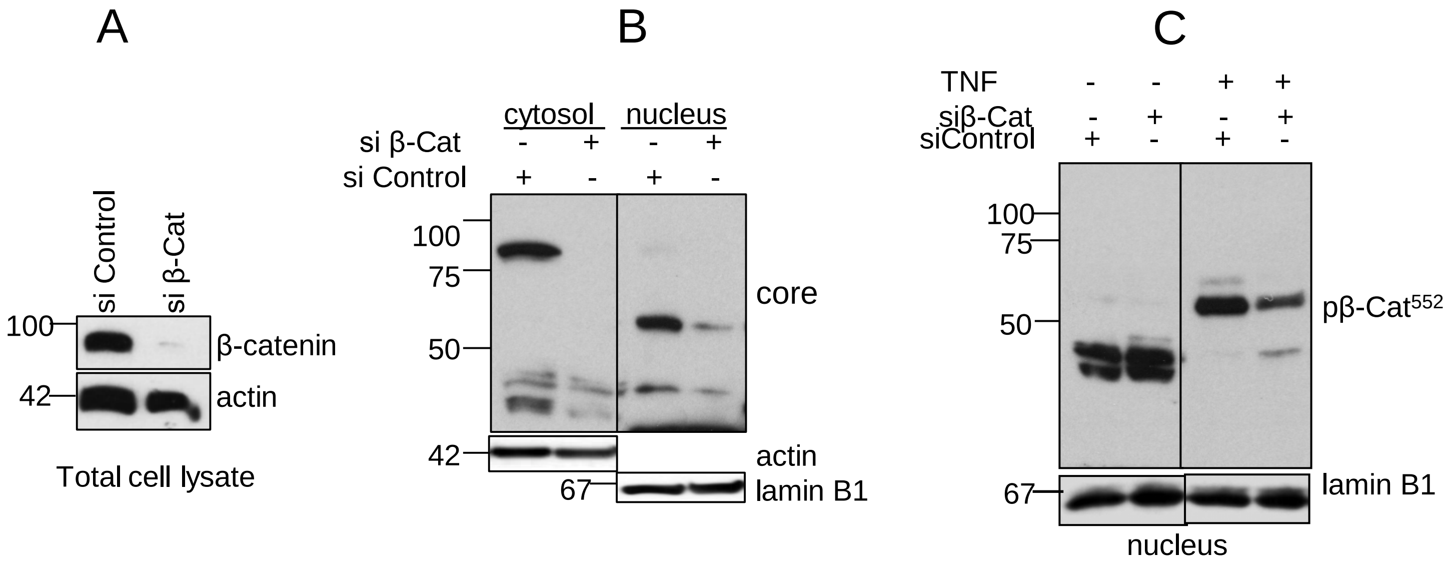

NCM460 cells were transiently transfected with siRNA to β-catenin.

A. β-catenin expression in total cell lysate detected by C-terminus specific antibodies.

B. β-catenin expression in cytosol and in nuclear lysates detected by core region specific antibodies. ***C.*** β-catenin expression in nuclear lysates stimulated by TNF detected by pβ-Cat^{552} specific antibodies. Actin in cytosol and lamin B1 in nucleus served as loading controls.

Supplemental Figure S3.

β-catenin truncation to LMW is proteasome dependent.

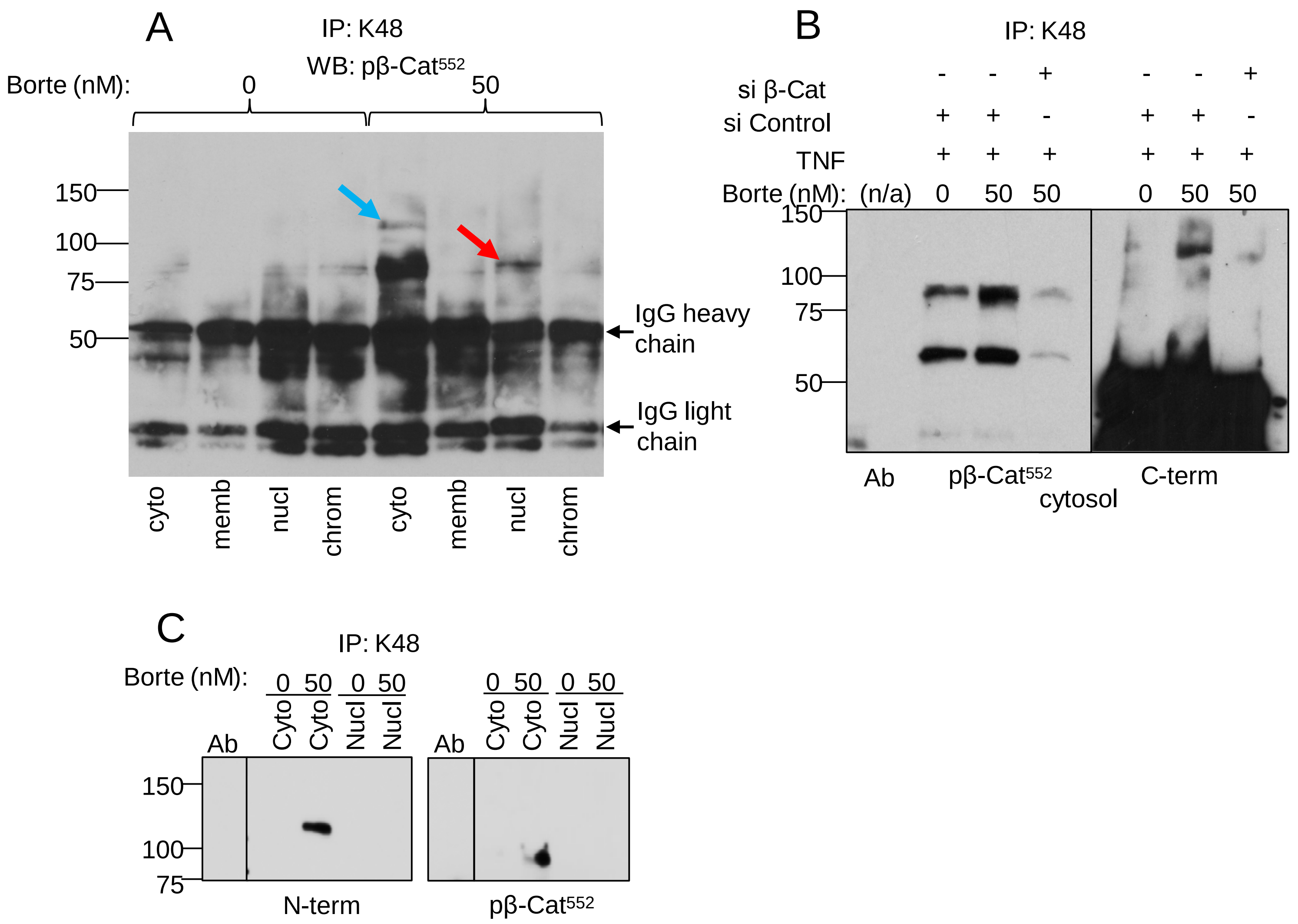

A. HT29 cells were treated with bortezomib (Borte) and fractionated to cytosolic (cyto), membranous (memb), soluble (nucl) and chromatin-bound (chrom) nuclear fractions. 500µg of protein from each fraction was immuniprecipitated for lysine 48 polyubiquitin chain (K48) and probed for pβ-Cat^{552}. Blue arrow indicates FS pβ-Cat^{552} with four ubiquitins attached , red arrow indicates ΔΔ pβ-Cat^{552} with four ubiquitins attached.

B. Cytosolic lysates from NCM460 cells, treated with Borte, TNF and siRNA as indicated, were immunoprecipitated by K48 and probed for C-terminal and pβ-cat^{552} antibodies.

C. Proteins from cytosolic and nuclear lysates from HT29 cells treated with Borte were denatured with TCA (Methods), immunoprecipitated by K48 and probed with N-terminal and pβ-Cat^{552} antibodies.

Supplemental Figure S4.

LMW bands of β-catenin are the result of proteasome-dependent degradation generated in cells prior to isolation.

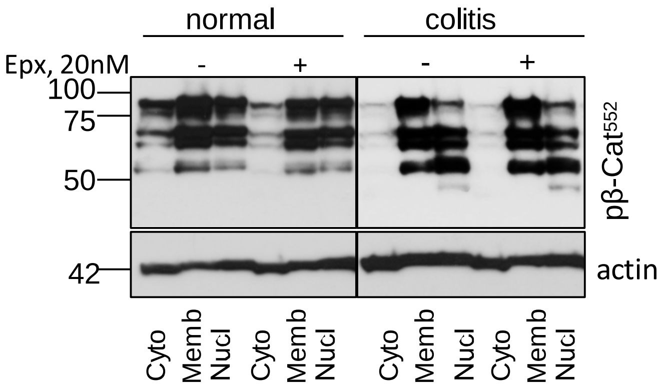

Human biopsies from normal and colitic patients were divided in half and fractionated into cytosolic, membrane and nuclear protein lysates. One half was fractionated in the presence of 20 nM epoxomicin. WB was probed for pβ-Cat^{552}. Actin indicates loading control.

Supplemental Figure S5.

Proteasome inhibitor reduces β-catenin transcriptional activity.

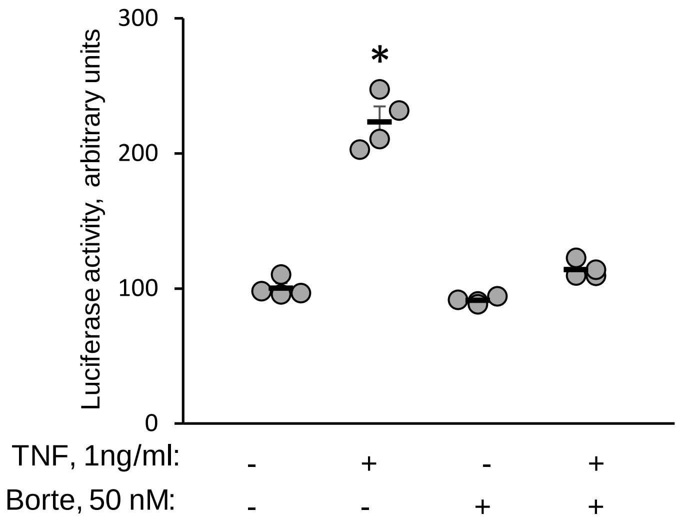

Luciferase assay of NCM460 cells transfected with TCF/LEF luciferase reporter construct. The cells were treated with TNF overnight and for 8hrs with bortezomib as indicated. *p<0.000015.

Supplemental Figure S6.

TCF4 binds LMW β-catenin.

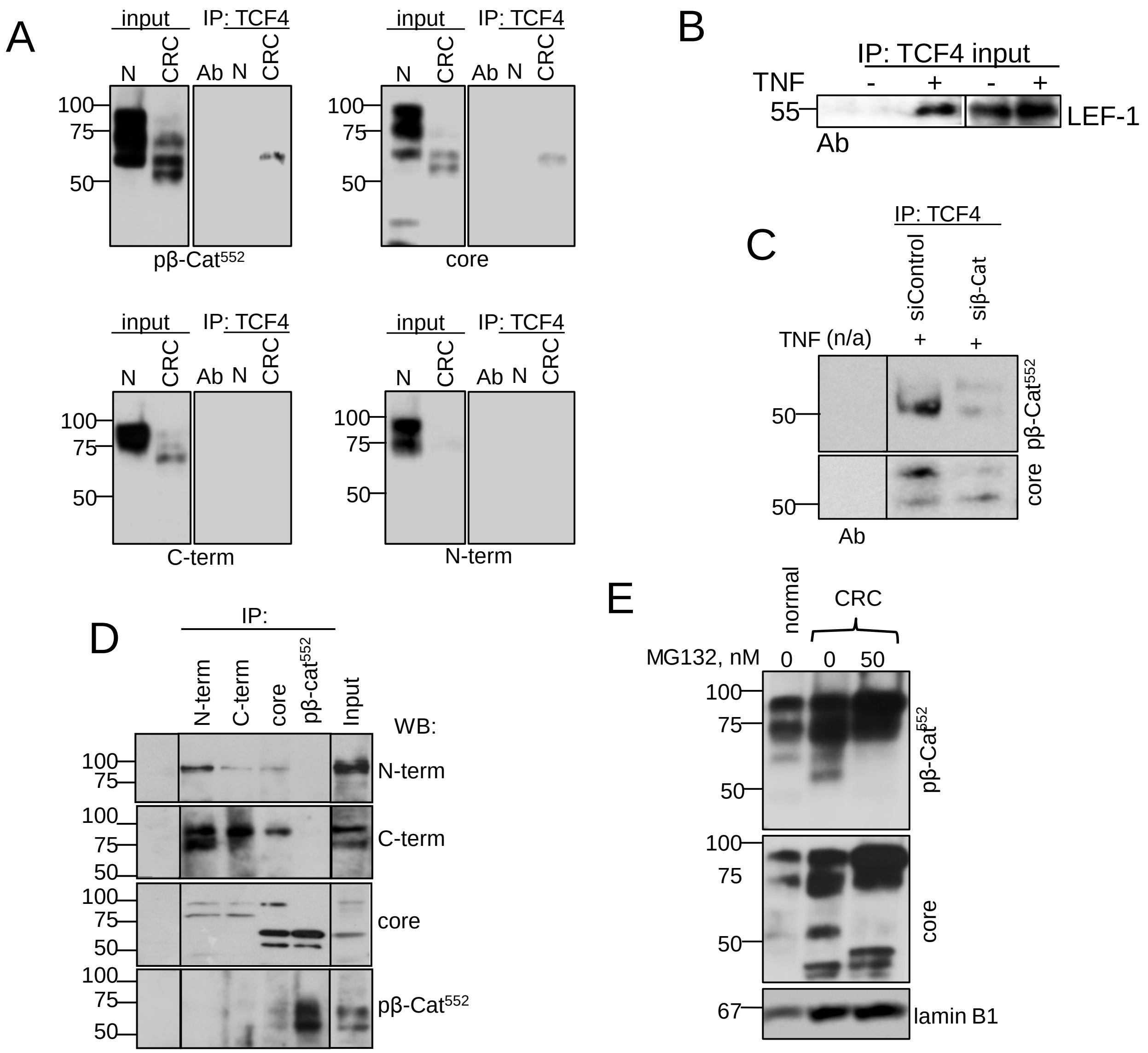

A. IEC nuclear fractions from normal and CRC biopsies were immunoprecipitated by anti-TCF4 and probed with β-catenin antibodies raised against different epitopes. ***B.*** Chromatin bound fraction from Fig.5A was immunoprecipitated by anti-TCF4 and probed for LEF-1. ***C.*** Nuclear fractions from NCM460 cells treated with TNF and siRNA as indicated were immunoprecipitated by anti-TCF4 and probed for pβ-Cat^{552} and core region specific antibodies. ***D.*** Nuclear protein fraction from CRC epithelial cells was used for immunoprecipitation with β-catenin antibodies indicated on the top. Precipitated proteins were probed with anti-β-catenin antibodies indicated on the right side. These are control WBs for the experiment presented in Fig. 4B. ***E.*** Nuclear fractions of normal, CRC and CRC treated with MG132 epithelial cells were probed with pβ-Cat^{552} and core region antibodies. Lamin B1 serves as a loading control. These are control WBs for the experiment presented in Fig. 4C.

Supplemental Figure S7.

LMW p-β-catenin is the dominant form in Wnt-treated mouse colonoids.

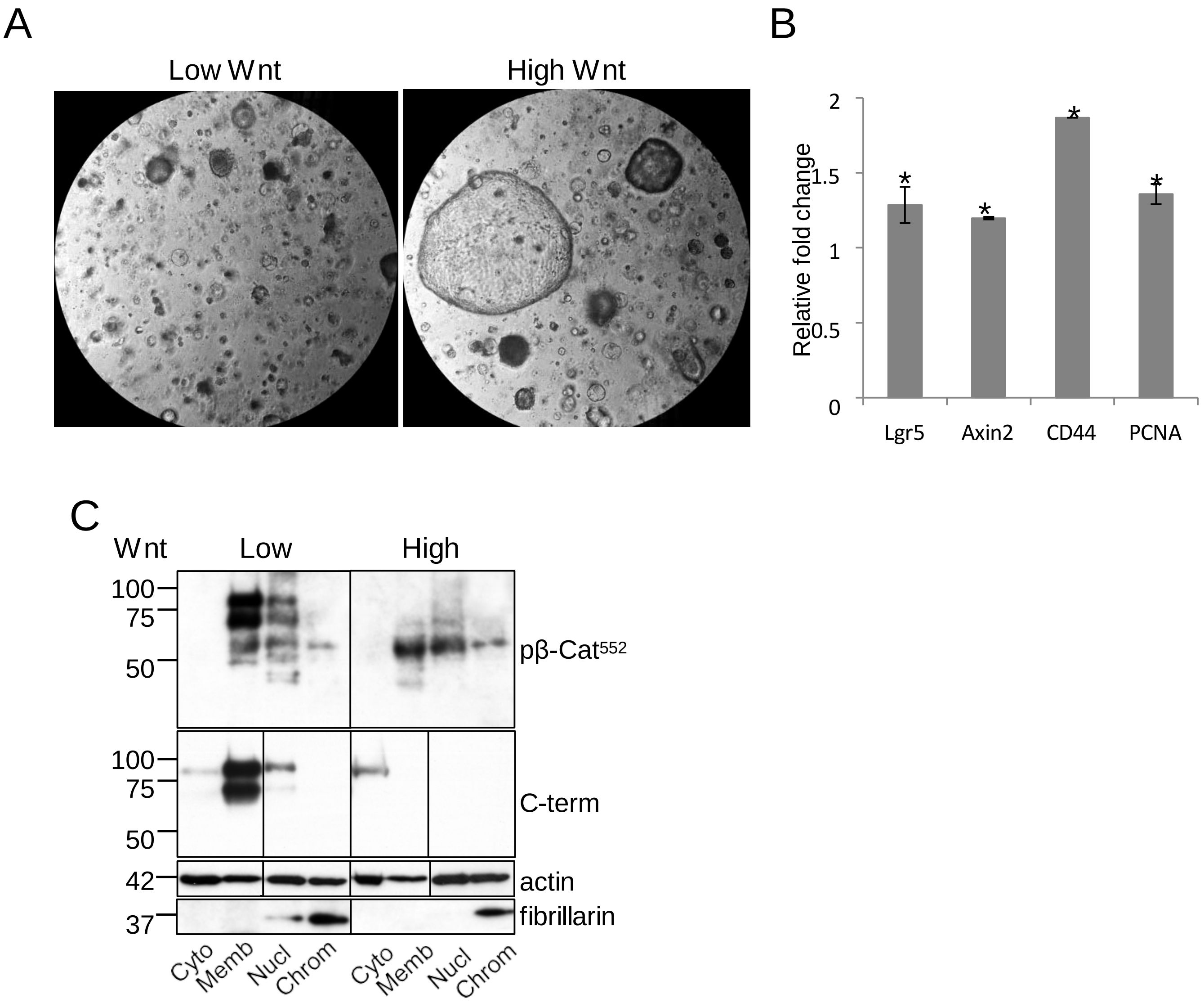

A. Bright field images of mouse colonoids grown in low or high Wnt conditions.

B. mRNA levels of Wnt target genes Lgr5, Axin2 and CD44, and proliferation marker PCNA in high Wnt colonoids relative to low Wnt. n=3 wells per experiment; *$p<0.05$.

C. WBs of colonoid fractions probed for pβ-Cat^{552} and C-terminal β-catenin. Actin and fibrillarin served as loading controls.

Supplemental Figure S8.

Overexpressed double truncated β-catenin localized in chromatin bound fraction enhances expression of β-catenin target genes.

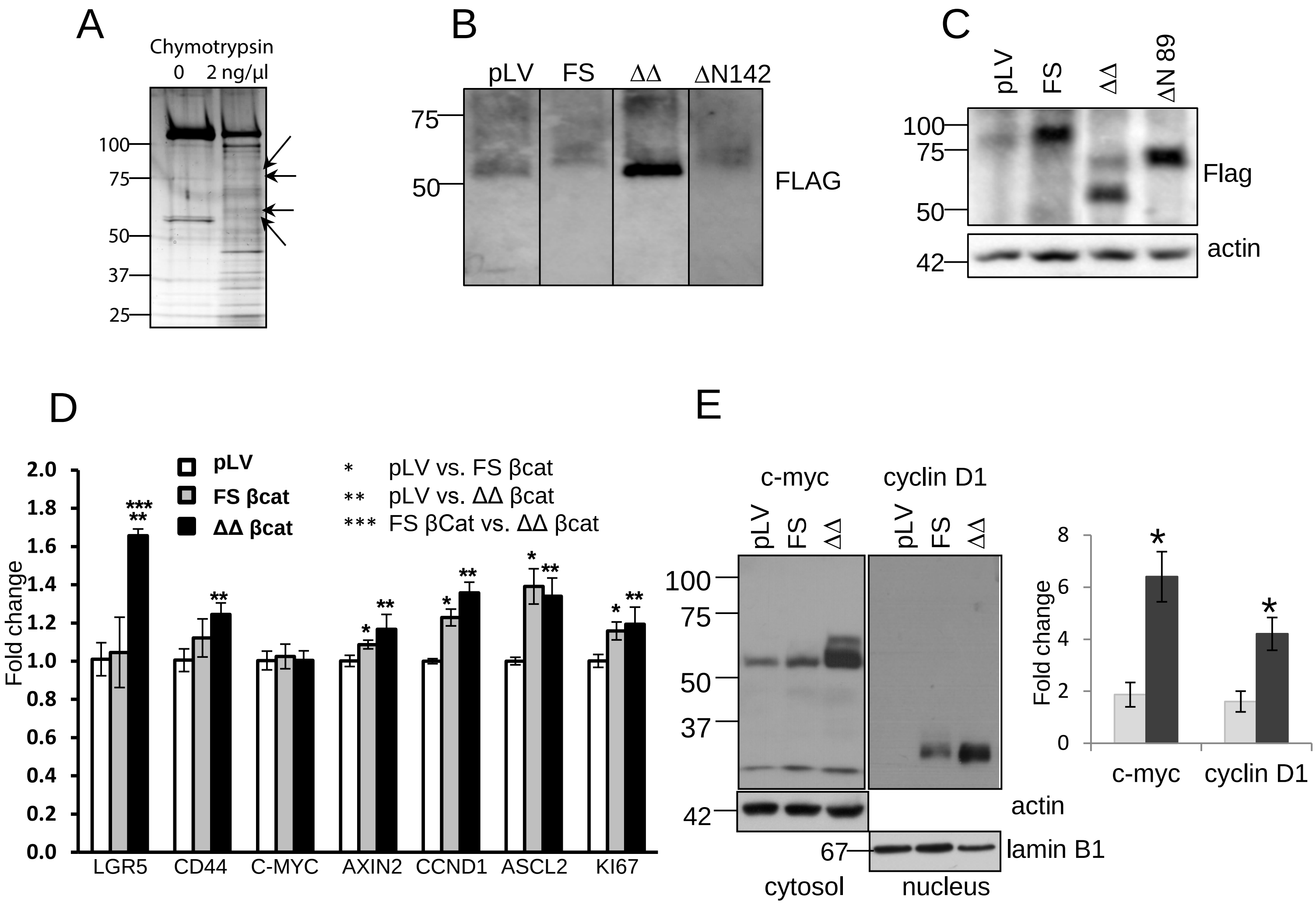

A. Silver staining of recombinant β-catenin cleaved with chymotrypsin. Left lane represents 5µg of un-cut β-catenin, the right lane shows 5µg of β-catenin incubated overnight with 2 ng/ml chymotripsin. Arrows show positions of 52-56 and ~75 kDa peptides.

B. Cobalt sepharose precipitates of nuclear chromatin bound extracts from pLV vector, FS, ΔΔ and Δ142 β-catenin overexpressing cell lines probed with anti-FLAG antibody.

C. Expression of FS, ΔΔ and Δ142 β-catenin in total lysates of infected NCM460 cells. WB for Flag tag. These lysates were used for experiments in Fig. 6B and C***.***

D. RT-PCR assay of β-catenin target genes in FS and ΔΔ β-catenin expressing NCM460 cell lines. n=4 wells per experiment; *$p \leq 0.029$, **$p \leq 0.05$, ***$p = 0.008$.

E. WBs for cytosolic c-myc and nuclear cyclin D1 in FS and ΔΔ β-catenin expressing NCM460 cell lines. Panel on right – densitometry data based on three independent experiments. *$p<0.05$.

Supplemental Figure S9.

Overexpressed double truncated β-catenin increases 3D culture colony formation and cell proliferation.

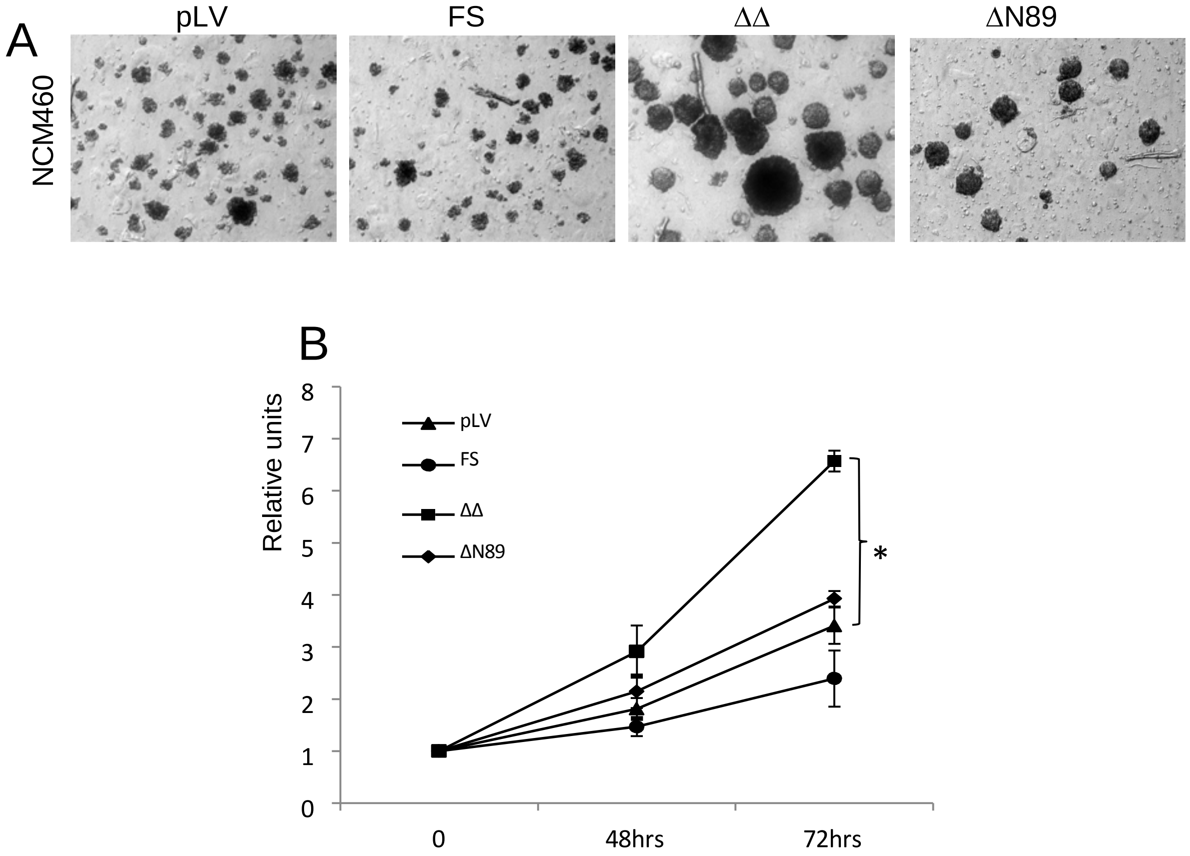

A. Methylcellulose colony formation assay for NCM460 cells infected with pLV vector, FS, ΔΔ and Δ89 β-catenin.

B. Proliferation test for cells used in A. *p<0.001.

Supplemental Figure S10.

Double-truncation and serine-552 phosphorylation of β-catenin increases tumor invasiveness.

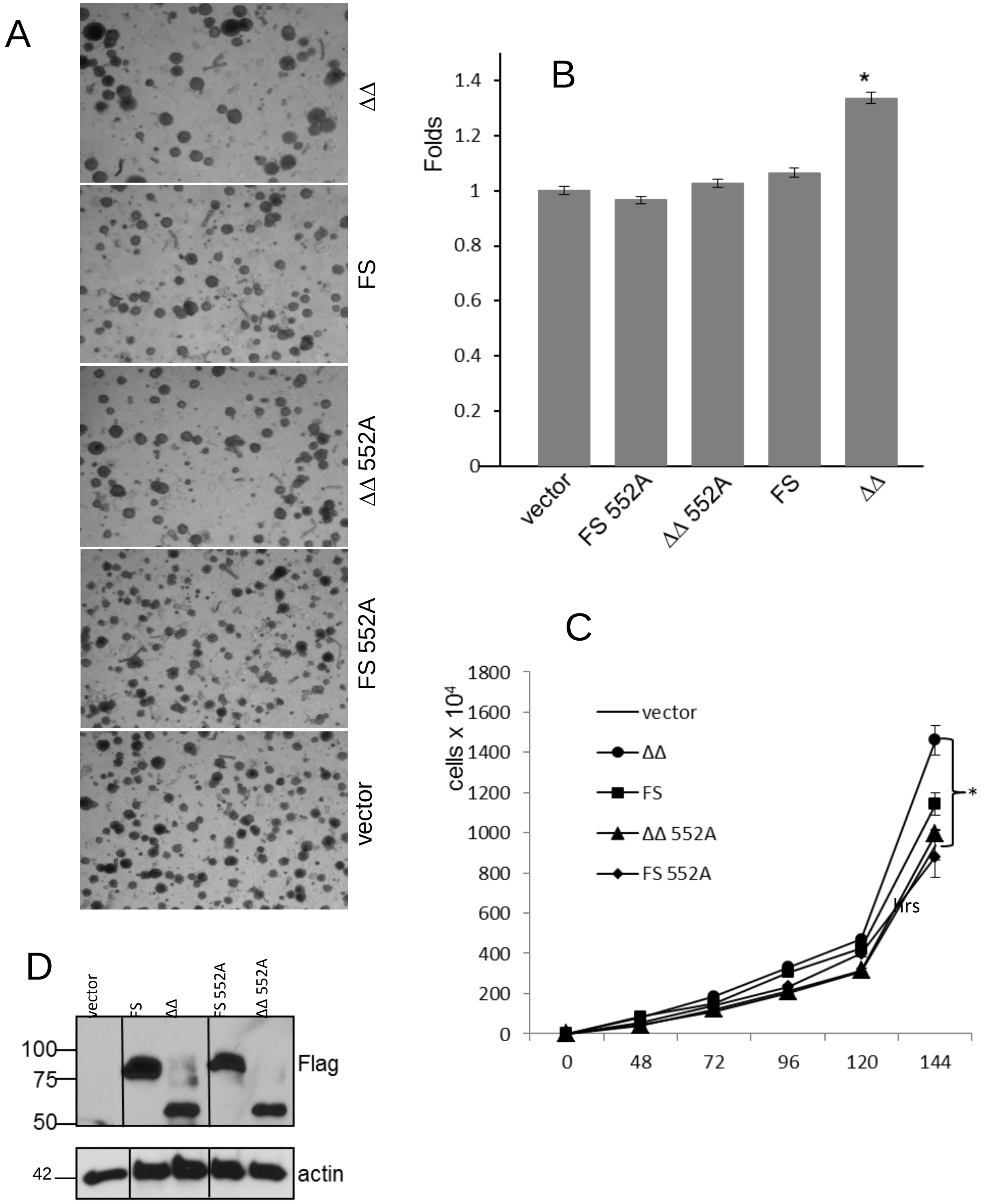

A. Methylcellulose colony formation assay for RKO cells transfected with FS, ΔΔ, FS552A and ΔΔ552A β-catenin.

B. Relative diameter of colonies presented in A. *p=0.003, n=300.

C. Proliferation test for cells used in A. *p=0.001 (vector vs. ΔΔ).

D. Flag-tagged β-catenin expression in total lysates of RKO cell lines used here and in Fig. 7.

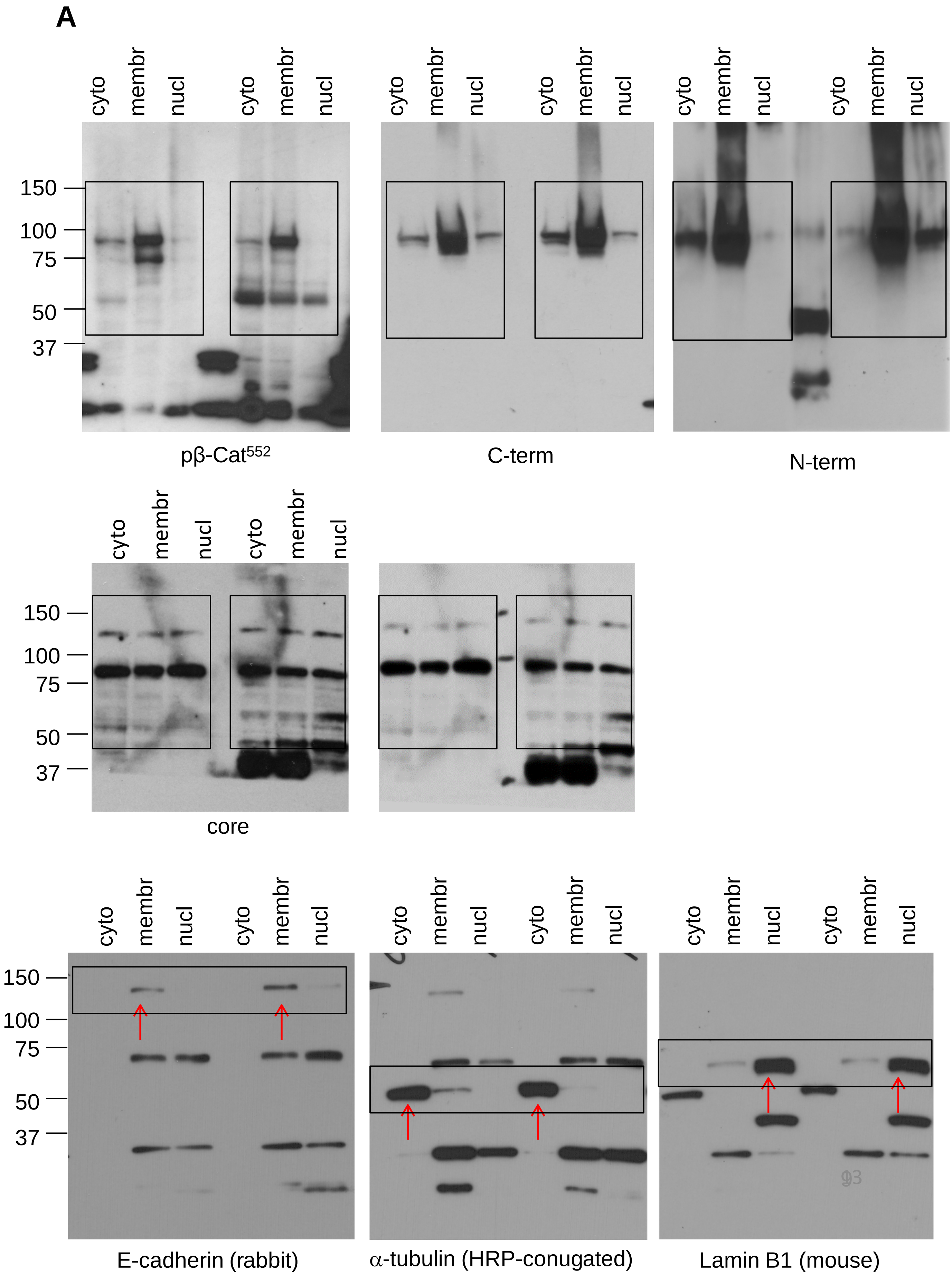
A
cyto
membr
nucl
cyto
membr
nucl
cyto
membr
nucl
cyto
membr
nucl
cyto
membr
nucl
cyto
membr
nucl
150
100
75
50
37
pβ-Cat552
C-term
N-term
cyto
membr
nucl
cyto
membr
nucl
150
100
75
50
37
core
cyto
membr
nucl
cyto
membr
nucl
cyto
membr
nucl
cyto
membr
nucl
cyto
membr
nucl
cyto
membr
nucl
150
100
75
50
37
E-cadherin (rabbit)
α-tubulin (HRP-conugated)
Lamin B1 (mouse)

Supplemental Figure SS1

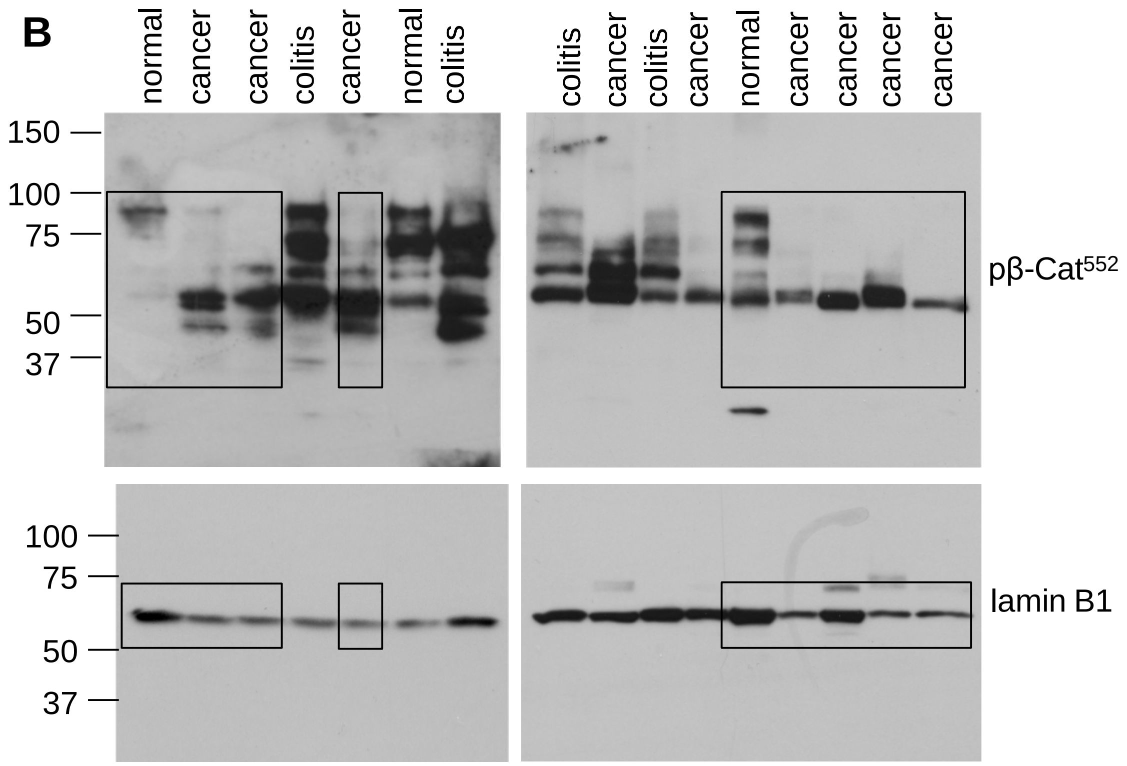

Supplemental Figure SS2

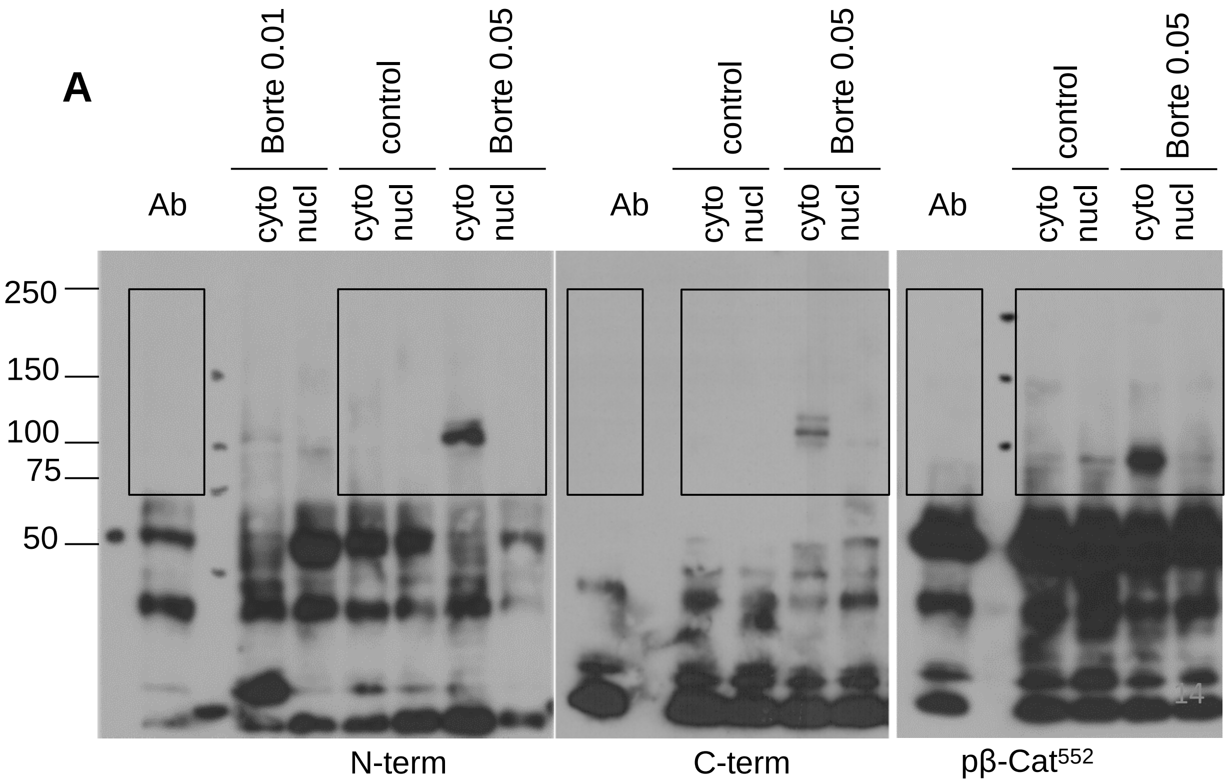

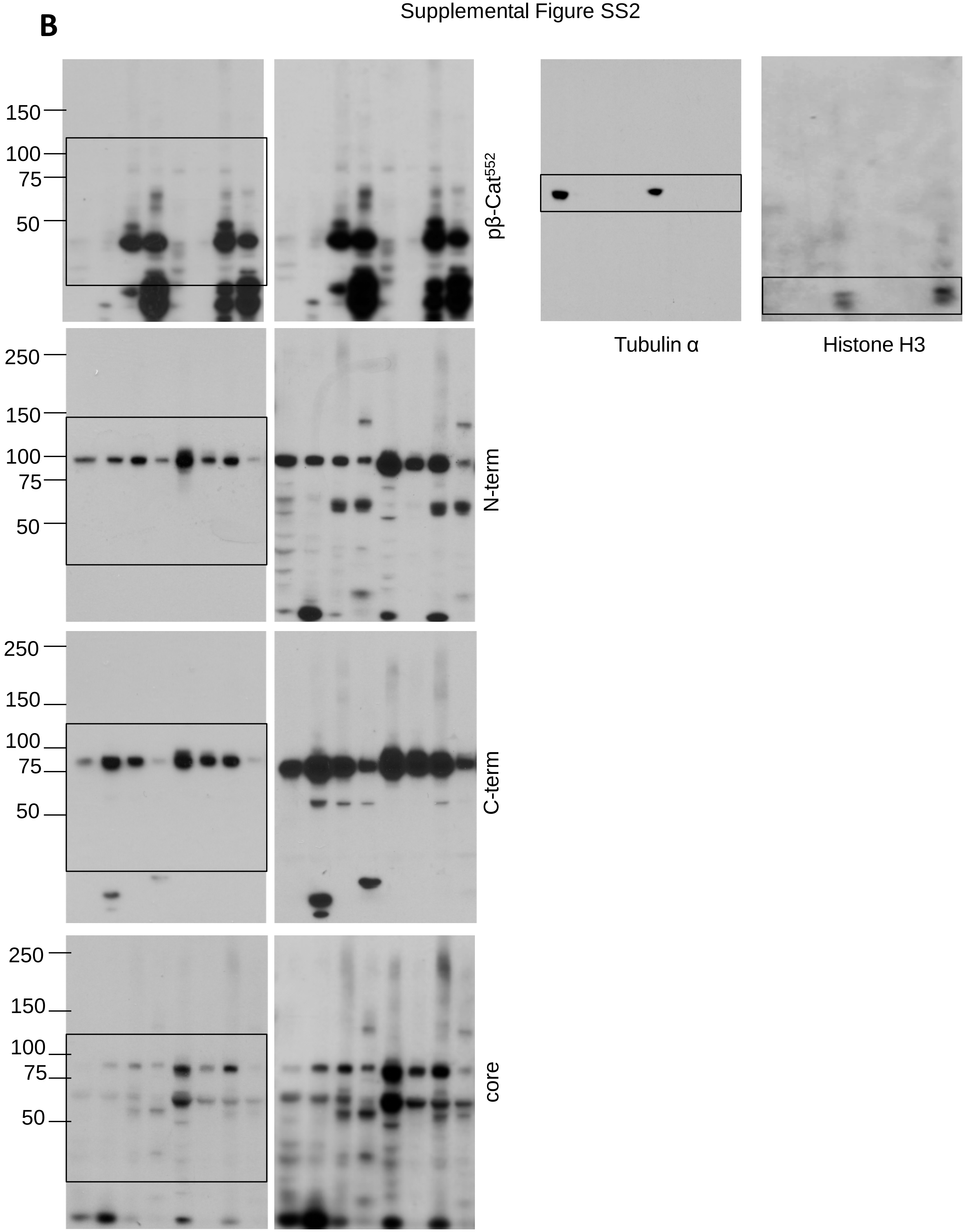
B
150
100
75
50
pβ-Cat552
250
150
100
75
50
N-term
250
150
100
75
50
C-term
250
150
100
75
50
core
Tubulin α
Histone H3

Supplemental Figure SS3

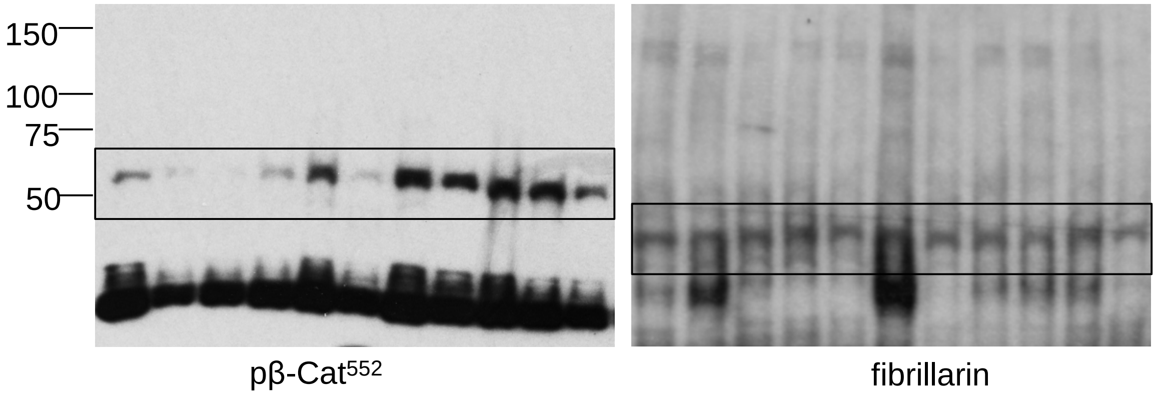

Supplemental Figure SS4

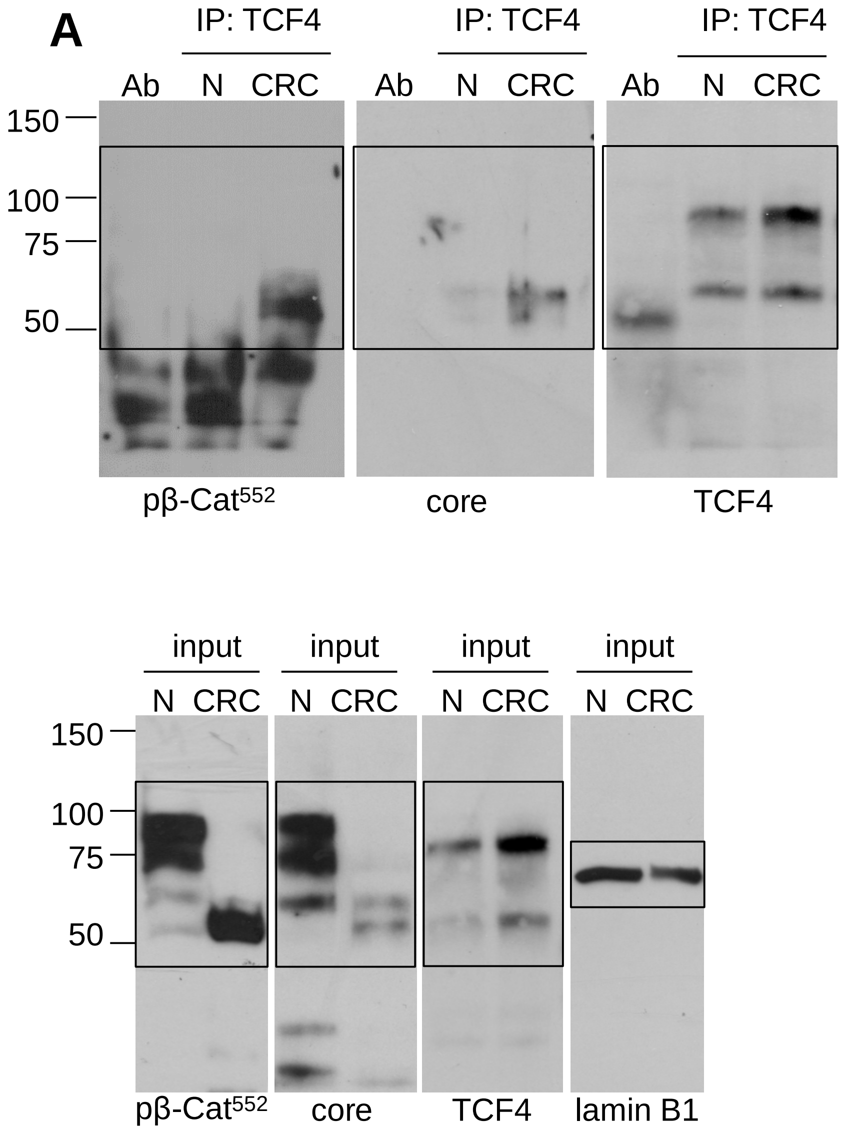

Supplemental Figure SS4

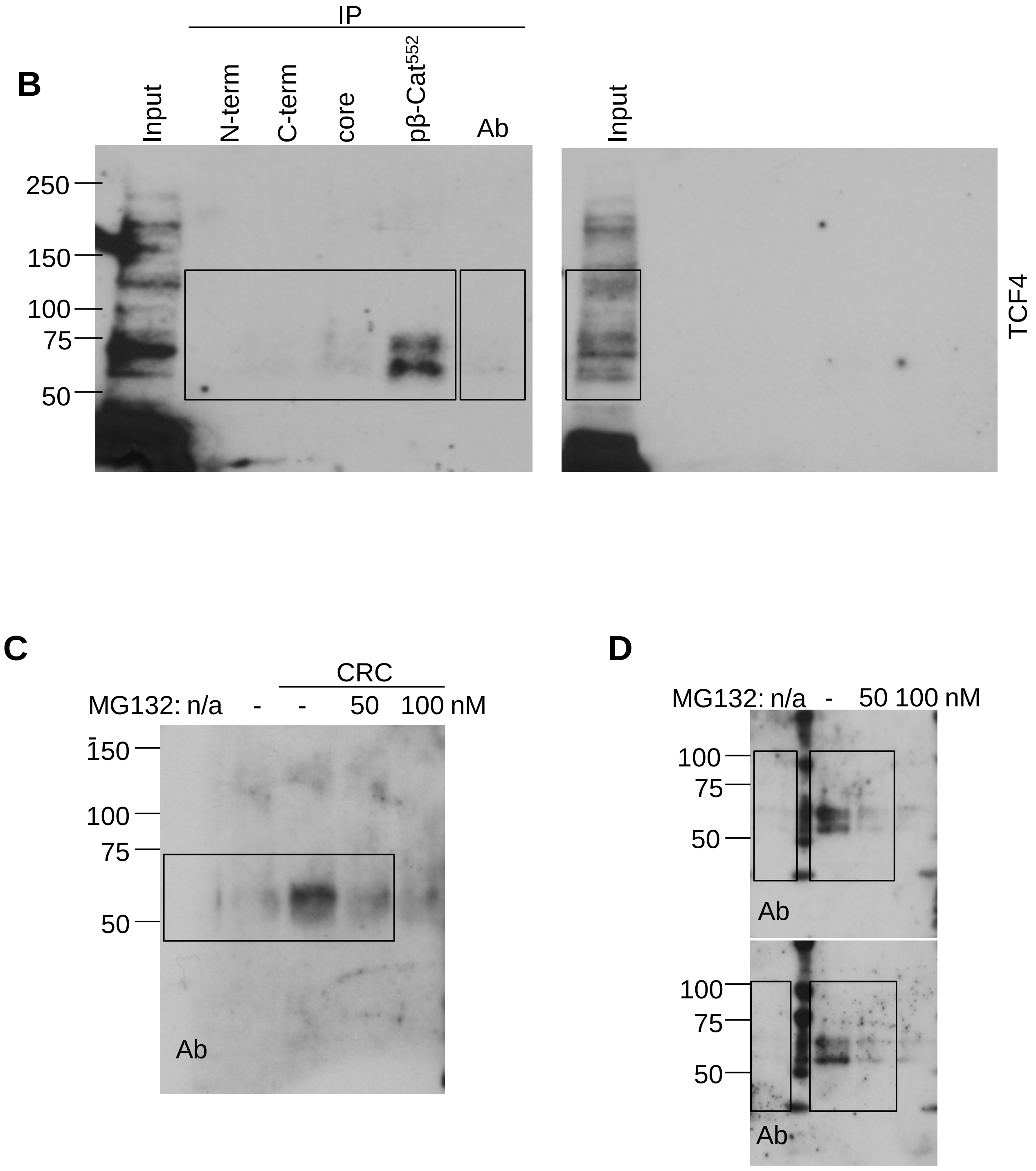

Supplemental Figure SS5

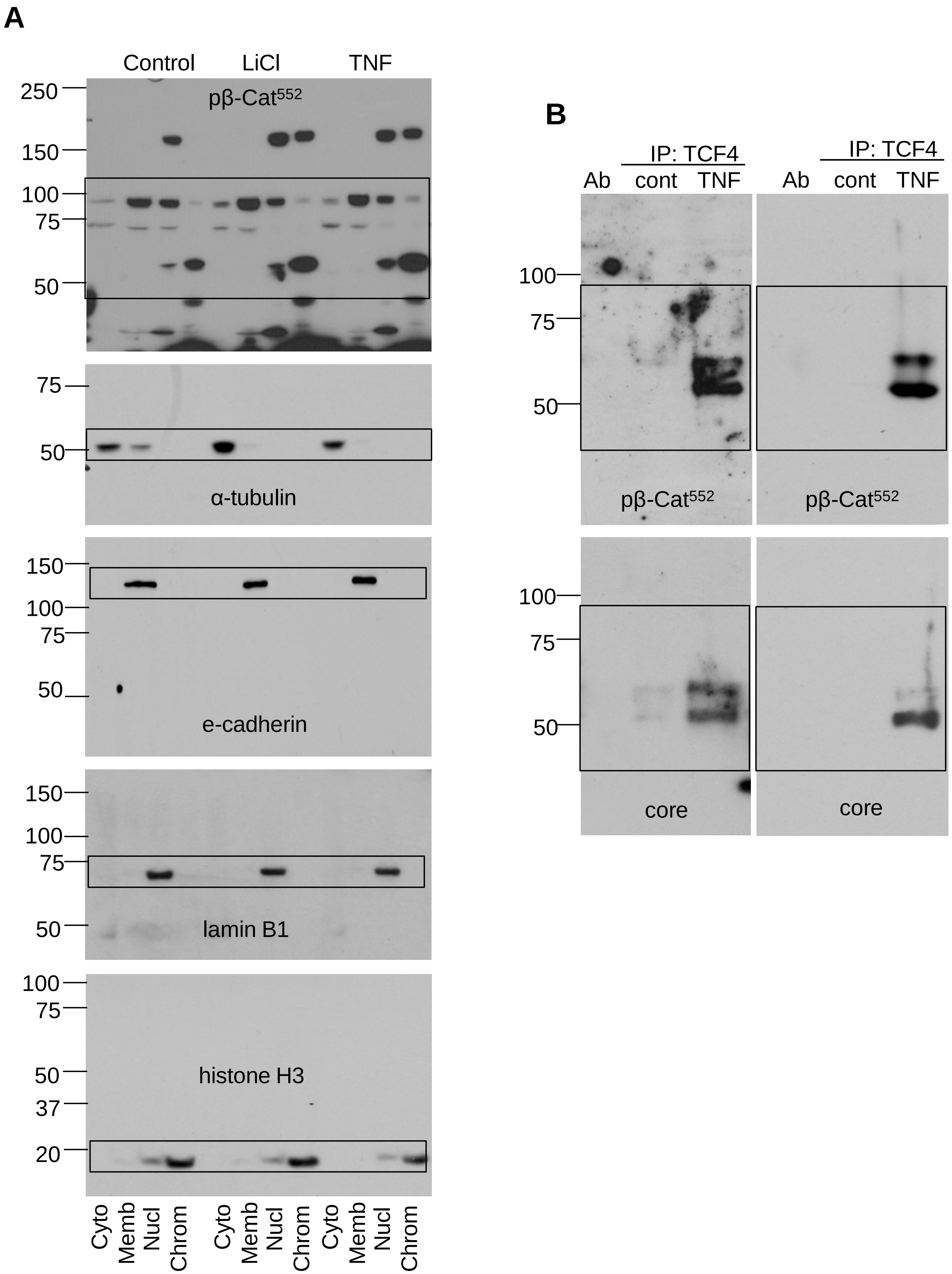

Supplemental Figure SS5

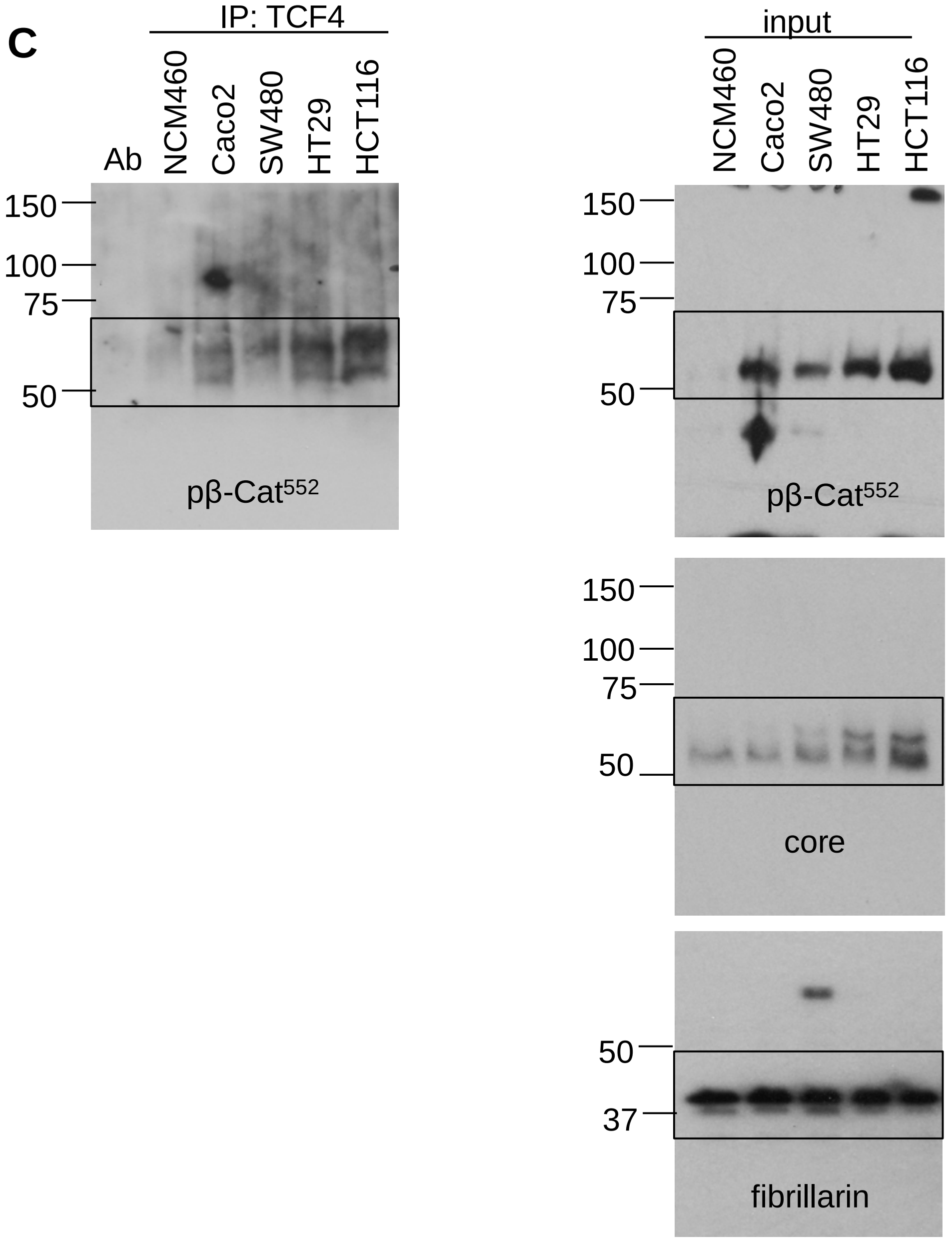

Supplemental Figure SS6

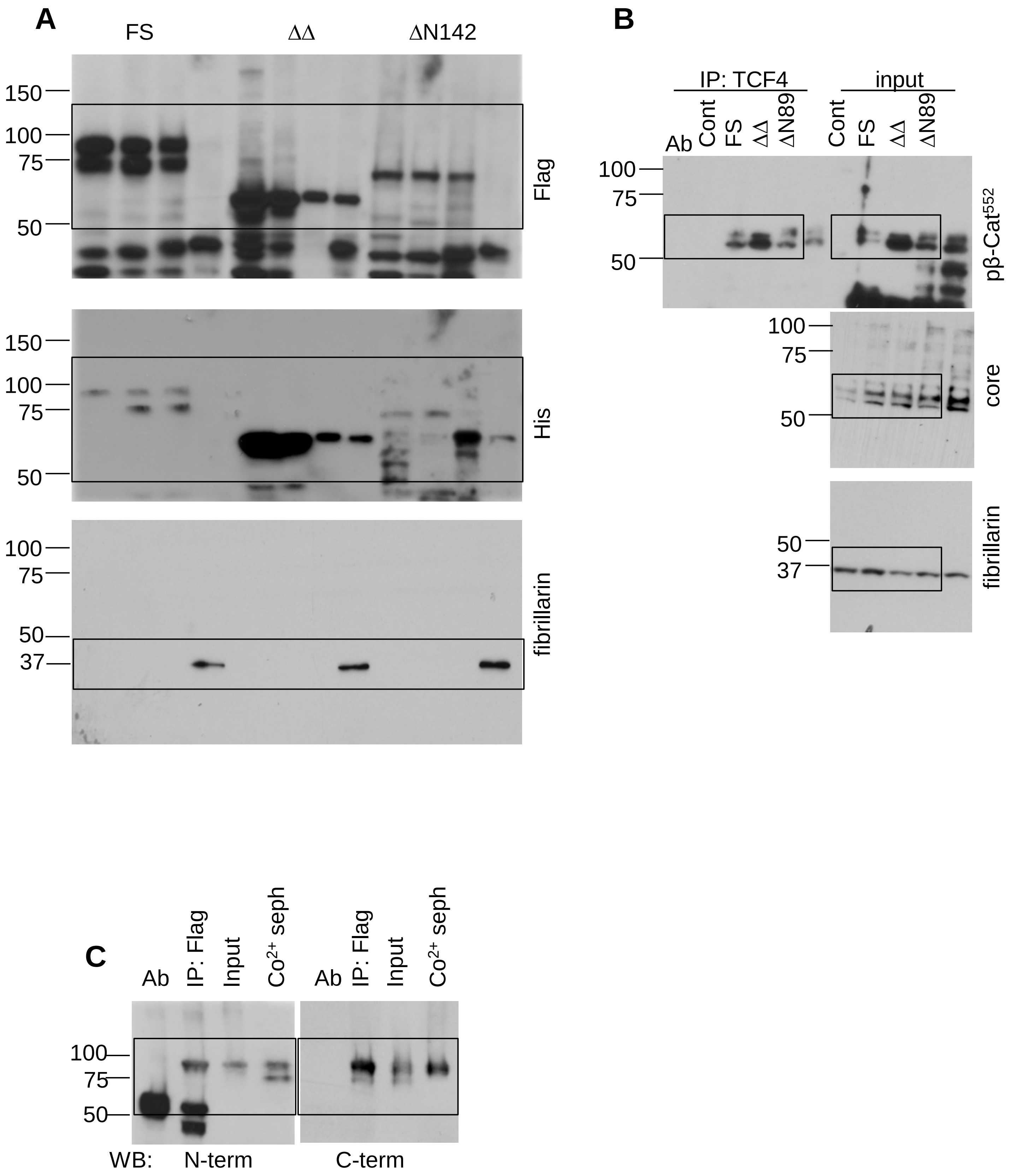

Supplemental Figure SS7

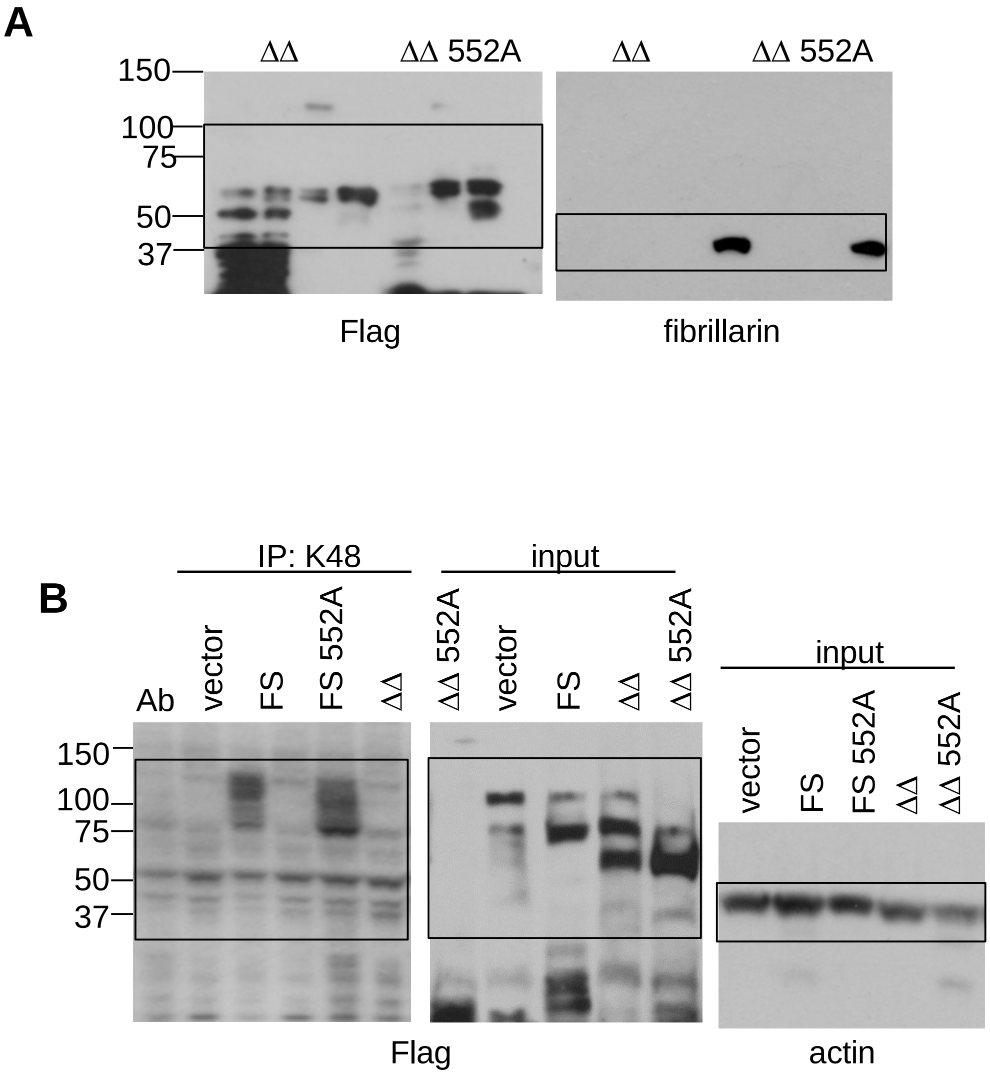

Supplement: Supplementary file 1 — Supplemental materials [file 41598_2017_18421_MOESM1_ESM.pdf]
